# Prophylactic Treatment of Probiotic and Metformin Mitigates Ethanol-Induced Intestinal Barrier Injury: *In Vitro*, *In Vivo*, and *In Silico* Approaches

**DOI:** 10.1155/2021/5245197

**Published:** 2021-09-27

**Authors:** Farhin Patel, Kirti Parwani, Priyashi Rao, Dhara Patel, Rakesh Rawal, Palash Mandal

**Affiliations:** ^1^Department of Biological Sciences, P.D. Patel Institute of Applied Sciences, Charotar University of Science and Technology, Changa, 388421 Anand, Gujarat, India; ^2^Department of Biochemistry & Forensic Science, University School of Sciences, Gujarat University, Ahmedabad, 380009 Gujarat, India

## Abstract

Ethanol depletes intestinal integrity and promotes gut dysbiosis. Studies have suggested the individual role of probiotics and metformin Met in protecting intestinal barrier function from injuries induced by ethanol. The objective of the current study is to investigate the potential mechanism by which coadministration of probiotic Visbiome® (V) and Met blocks the ethanol-induced intestinal barrier dysfunction/gut leakiness utilizing Caco-2 monolayers, a rat model with chronic ethanol injury, and in silico docking interaction models. In Caco-2 monolayers, exposure to ethanol significantly disrupted tight junction (TJ) localization, elevated monolayer permeability, and oxidative stress compared with controls. However, cotreatment with probiotic V and Met largely ameliorated the ethanol-induced mucosal barrier dysfunction, TJ disruption, and gut oxidative stress compared with ethanol-exposed monolayers and individual treatment of either agent. Rats fed with ethanol-containing Lieber-DeCarli liquid diet showed decreased expression of TJ proteins, and increased intestinal barrier injury resulting in pro-inflammatory response and oxidative stress in the colon. We found that co-administration of probiotic V and Met improved the expression of intestinal TJ proteins (ZO-1 and occludin) and upregulated the anti-inflammatory response, leading to reduced ER stress. Moreover, co-administration of probiotic V and Met inhibited the CYP2E1 and NOX gene expression, and increase the translocation of Nrf-2 as well as anti-oxidative genes (SOD, catalase, Gpx, and HO-1), leading to reduced colonic ROS content and malondialdehyde levels. The combined treatment of probiotic V and Met also improved their binding affinities towards HO-1, Nrf-2, SLC5A8, and GPR109A, which could be attributed to their synergistic effect. Our findings based on *in-vitro*, *in-vivo*, and *in-silico* analyses suggest that the combination of probiotic V and Met potentially acts in synergism, attributable to their property of inhibition of inflammation and oxidative stress against ethanol-induced intestinal barrier injury.

## 1. Introduction

Chronic alcohol consumption is associated with numerous gastrointestinal and liver disorders, especially alcoholic liver disease (ALD) [1]. While the precise molecular mechanism of ALD pathogenesis is not fully understood, few of the reports suggested that ethanol-induced barrier dysfunction plays a crucial role in ALD progression [2, 3]. Intestinal epithelial tight junctions (TJs) are considered to be the key regulators of intestinal mucosal permeability [4]; therefore, regulating the TJ protein expression (i.e., occludin, claudins, and zonula occludens (ZO)) or modulating its functions will positively influence intestinal barrier function. Alcohol consumption is known to disrupt TJs resulting in increased intestinal permeability [5]. It is now a most likely accepted fact that impaired intestinal epithelial integrity and intestinal barrier dysfunction are the two principle reasons behind the increased intestinal permeability [6]. Studies have indicated that alcohol-induced increased intestinal permeability leads to an anomalous leakage of bacterial endotoxins, thereby causing liver damage [7]. In alcoholics, increased blood endotoxin levels (endotoxemia) can be due to three potential mechanisms: (i) due to both abnormal gut dysbiosis and overproduction of bacteria leading to increased endotoxin production; (ii) due to gut leakiness, which causes increased endotoxin permeation through the gut; and (iii) due to either portal hypertension or Kupffer cell dysfunction leading to decreased endotoxin elimination [8, 9]. When there is no evidence of portal hypertension and defective Kupffer's cell function, then it is not considered as an important factor in the initiation of ALD [8]. Therefore, gut leakiness or disrupted intestinal barrier function is considered to be the important mechanism for ethanol-induced increased endotoxemia in alcohol-mediated liver injury [10].

Equally unclear is the molecular mechanism mediating ethanol-induced gut leakiness. Several lines of evidence suggest that the culprit is ethanol-induced tissue oxidative stress. Studies showed that alcohol disrupts the barrier integrity of monolayers of intestinal cells and that alcohol-induced disruption is due to oxidative injury to the cytoskeleton [11]. CYP2E1, an enzyme playing an important role in alcohol metabolism, may contribute to alcohol-induced intestinal effects [12]. Oxidative stress and ROS are predominantly generated through the induction of CYP2E1 metabolism that may perhaps facilitate the disruption of intestinal permeability [13]. The challenge to treat any stage of ALD other than by abstinence still lies in the incapability to recognize new therapeutic strategies. Therefore, studies showed that a better understanding of the exact molecular mechanisms responsible for the ethanol-induced increase in intestinal permeability may eventually result in more effective prevention/treatment strategies for inhibiting the progression of ALD [14].

The intestinal bacterium that refers to gut microbiota has a reflective impact on the host metabolism and its immune system [15]. Indeed, intestinal bacteria contribute to the expansion of the intestinal architecture and immune system and also play a lead role in gut barrier function maintenance [16]. Among all the beneficial functions of gut microbiota, its capability to ferment long-chain polysaccharides that avoid human metabolism producing short-chain fatty acids (SCFAs) is considered to be the best characteristic. The predominant SCFAs produced are acetate, propionate, and butyrate [17]. Among the three SCFAs, butyrate is present to a smaller extent, but is the most effective. After ethanol consumption, the SCFA levels in the intestine were noted to be decreased, except for acetate, whose levels are known to be increased as it is the metabolite of ethanol [18].

Modulation of gut microbiota through probiotic administration is considered to be one of the optimized formulations towards alcohol-induced gut injury. According to a World Health Organization (WHO)/Food and Agriculture Organization (FAO) report, probiotics are live microorganisms that when used in suitable quantities, confer a health benefit on the host improving the functional properties of gut microbiota [19]. In the past years, emerging concepts for the use of probiotics as functional products have been observed in preventing the ethanol-induced disruption of colonic epithelial TJs and barrier dysfunction [20]. Additionally, an *in vivo* study explained that the probiotic *Lactobacillus rhamnosus* GG (LGG) helps in reducing gut permeability, oxidative stress, and inflammation in the liver and intestine [21].

Met is another such medicinal compound known to possess anti-hyperglycemic activity that has a potential to prevent alcohol-induced liver injury [22]. Recent studies have revealed that (Met) exerts a constructive role in attenuating intestinal dysbiosis. In type 2 diabetes, Met has been known to alleviate the gut microbiota imbalance partially [23]. Studies also demonstrated the role of Met in preventing the intestinal mucosal barrier injury and inflammation caused by dextran sulfate sodium (DSS) [24]. In LPS-induced *in-vitro* and *in vivo* models, Met alleviates the intestinal TJ dysfunction, oxidative stress, and inflammatory response [25].

Since intestinal injury is associated with oxidative stress and free radical formation, compounds with antioxidant properties could act as an inhibitor in ameliorating ethanol-induced intestinal injury. Considering the above experimental studies as well as the individual protective role of probiotics and Met towards intestinal homeostasis, the current study was executed to investigate the potential combinatorial role of probiotic V and Met in preventing the increased intestinal barrier integrity, epithelial cell permeability, and butyrate levels through interactions with receptors and transporters by inhibiting the gut oxidative stress and inflammation in ethanol-induced intestinal injury (*in vitro* and *in vivo* models of intestinal injury). To know and understand the plausible mechanism by which combined treatment of probiotic V and Met could act in synergism to prevent oxidative stress and to maintain intestinal permeability induced by ethanol, we adopted an in silico approach and performed the docking of Met and butyrate (metabolite released from probiotic V) with antioxidants, i.e., Nrf-2 and HO-1 as well as butyrate sensors, i.e., the butyrate receptor GPR109A and the butyrate transporter SLC5A8.

## 2. Materials and Methods

### 2.1. Materials

All chemicals were purchased from HiMedia Laboratories (HiMedia Laboratories Private Limited, India) unless cited otherwise. The TRIzol Reagent, cDNA Synthesis Kit, and SYBR/ROX Master Mix were purchased from Thermo Fisher Scientific (USA). The metformin reagent and primers for quantitative real-time reverse transcription-polymerase chain reaction (qRT-PCR) were obtained from Sigma-Aldrich (USA).

### 2.2. Cell Culture

In the present study, a human colonic epithelial cell line, i.e., Caco-2 cells, were acquired from the National Centre for Cell Sciences (NCCS), Pune, India, and were cultured in dulbecco's modified eagle medium (DMEM) medium supplemented with 20% (*v*/*v*) fetal bovine serum (FBS) and 1% (*v*/*v*) antibiotic-antimycotic solution in a humidified atmosphere of 5% (*v*/*v*) CO_2_ at 37°C. The experimental dose of probiotic V and Met (i.e., 10 *μ*l and 1 mM, along with 100 mM ethanol) was previously determined in our lab using an MTT cytotoxicity assay [26]. Cells were maintained in T75 flasks, and the medium was replaced every second day for 23 days. After attaining 70% confluency, cells were detached by using a 0.25% trypsin-EDTA solution for about 5 min, then centrifuged at 1000 rpm for 5 min, removed the supernatant, resuspended in 16 mL of complete medium (DMEM), and seeded equally into two new T75 flasks. Cells from passages 30 to 38 were used for all experiments.

### 2.3. Probiotic Visbiome® Supplementation in an *In Vitro* and *In Vivo* Model of Intestinal Barrier Injury

Visbiome® (Lot #07197721) is considered to be a probiotic mixture of three viable lyophilized bacterial species containing 112.5 × 10^9^ CFU/capsule. The consortium includes four strains of lactobacilli (*Lactobacillus acidophilus*, *Lactobacillus paracasei*, *Lactobacillus delbrueckii* subspecies *bulgaricus*, and *Lactobacillus plantarum*), three strains of bifidobacteria (*Bifidobacterium longum*, *Bifidobacterium infantis*, and *Bifidobacterium breve*), and *Streptococcus salivarius* subspecies *thermophilus*. Probiotic Visbiome® (V) containing 1 g of each stock was suspended in the De Man, Rogosa, and Sharpe agar (MRS) broth (pH: 6.5 ± 0.2) at 25°C to activate the probiotic V culture. The probiotic V stock culture was conserved through a glycerol-MRS broth at a concentration of 20% (*v*/*v*) and maintained at -70°C.

#### 2.3.1. For *In Vitro* Supplementation

The probiotic V culture was sonicated for 30 min (repeating 10 s of sonication and 10 s of hold) with a sonicator. The probiotic V bacterial culture at its respective final concentration was centrifuged at 1500 × g for 10 min and the individual supernatant (whole-cell extract) was obtained. Furthermore, the obtained supernatant was centrifuged at 6500 × g for 30 min yielding the cell cytosol (supernatant) and the membrane (pellet). The cell-free supernatant was stored at -20°C and used for further experimentation.

#### 2.3.2. For *In Vivo* Supplementation

The probiotic V (1%) culture was inoculated into another freshly prepared MRS broth. The probiotic V inoculum was collected by centrifugation at 10,000 × g and 4°C for 10 min. After centrifugation, the remaining cell pellet was washed thrice with phosphate-buffered saline (PBS). Then, the cell pellet was resuspended in sterile distilled water with 10^8^ colony-forming unit (CFU)/ml as a final concentration for further experimental use.

### 2.4. Animals

Eight- to ten-week-old male Wistar rats weighing 200-225 g were acquired from Zydus Pharmaceutical Industries Pvt. Ltd. (India). Rats were allowed to acclimatize in standard cages (two rat/cage) and were fed a normal chow diet under ambient conditions (temperature: 22 ± 2°C; relative humidity: 55 ± 5%) with a 12 h light and 12 h dark cycle before the commencement of the feeding experiment with the Lieber DeCarli liquid diet.

### 2.5. *In Vitro* and *In Vivo* Induction of Intestinal Barrier Injury Using Ethanol Stimulation with Simultaneous Probiotic Visbiome® and Metformin Administration

#### 2.5.1. *In Vitro* Model

Caco-2 cells were seeded in 6-well plates at the seeding density of 1.8 × 10^5^ cells/ml and cultured for 23 days. Later, we assigned Caco-2 monolayers into the following groups: (a) the control group of untreated Caco-2 monolayers; (b) the ethanol group—cells were treated with serum-free 100 mM ethanol-containing medium for 48 h to induce intestinal epithelial barrier dysfunction; (c) experimental group-1—cells were treated with serum-free medium containing 1 mM Met concentration along with 100 mM ethanol for 48 h; (d) experimental group-2—cells were treated with serum-free medium containing 10 *μ*l/ml probiotic V along with 100 mM ethanol for 48 h; and (e) experimental group-3 with the combination—cells were treated with serum-free medium containing 1 mM Met and 10 *μ*l/ml probiotic V along with 100 mM ethanol for 48 h.

#### 2.5.2. *In Vivo* Model

Weight and age-matched rats were given Liber DeCarli's diet for the first 2 days for acclimatization. After 2 days, ethanol-fed rats were permitted free access to a complete ethanol-containing Lieber DeCarli diet. Also, control rats were given a pair-fed diet containing maltodextrin (substituted isocalorically) for the entire feeding period. The ethanol-induced intestinal barrier injury model (25 days, 32% total calories) contained increased concentrations of ethanol (vol/vol): 1% (2 days), 2% (2 days), 4% (7 days), 5% (7 days), and lastly 6% (7 days) [26].

Oral supplementation of probiotic V and Met was given at doses of 10^8^ CFU/day and 75 mg/kg, respectively, to the experimental rats [26]. Following ethanol exposure protocols, rats were randomized, weighed, and anesthetized. Following feeding protocols, blood was taken from the posterior vena cava, and from the part of whole blood, serum was isolated and kept at -80°C, until further use. Moreover, rats fasted overnight were then euthanized by the exsanguination method (as mentioned in the CPCSEA guidelines) and colons were excised. Other small portions of the colon were fixed in formalin, frozen in optimal cutting temperature (OCT) medium, and stored in RNA later at -20°C for isolating RNA.

Therefore, to induce the intestinal barrier injury rat model, we assigned rats to the following groups: (a) the control group—rats fed with a pair-fed diet containing maltodextrin (substituted isocalorically); (b) the ethanol control group—rats fed with the ethanol-containing Lieber DeCarli liquid diet for 25 days to induce intestinal barrier dysfunction; (c) experimental group-1—rats fed with 75 mg/kg body weight Met along with the ethanol containing Lieber DeCarli liquid diet for 25 days; (d) experimental group-2—rats fed with 10^8^ CFU/day Probiotic V along with the ethanol containing Lieber DeCarli liquid diet for 25 days; and (e) experimental group-3 with the combination—rats fed with 75 mg/kg body weight Met and 10^8^ CFU/day Probiotic V along with the ethanol-containing Lieber DeCarli liquid diet for 25 days.

### 2.6. Measurement of Transepithelial Electrical Resistance (TEER) in Caco-2 Monolayers

To evaluate the intestinal barrier integrity, Caco-2 monolayers with a seeding density of 1.0 × 10^5^ cells/transwell were seeded in 12-well plates. The respective treatments given are based on the groups described earlier, and the percentage of TEER was measured. 0.5 ml and 1.5 ml media were added to the upper and lower chambers, respectively. The TEER value was measured by an epithelial volt ohmmeter. For blanks, inserts without cells were considered and their mean resistance was deducted from all control and treated samples. Triplicate measurements were recorded for each monolayer, and the average value was calculated to measure electrical resistance. TEER was calculated as follows: TEER = (Rm–blank) × *A*; where Rm = transmembrane resistance; blank = intrinsic resistance of a cell‒free media; and *A* = membrane surface area (cm^2^) [27].

### 2.7. Determination of *In Vitro* and *In Vivo* Intestinal Permeability

#### 2.7.1. *In Vitro* Model

To measure the intestinal epithelial barrier permeability, fluorescein isothiocyanate- (FITC-) dextran (FD-4), a paracellular marker, was chosen, and the amount of the marker that passes the Caco-2 cell monolayers was measured. Briefly, Caco-2 cells were seeded in 12-well plates at a density of 1.0 × 10^5^ cells/well for 21 days. The respective treatments given are based on the groups described earlier. Afterward, FD-4 was added to the apical compartment of the Caco-2 monolayer transwell inserts and incubated for 30 min. The FD-4 outflow into the lower basal compartment was measured at the excitation wavelength of 485 nm and an emission wavelength of 530 nm using a fluorescence spectrophotometer (Perkin Elmer LS-55, USA) [28].

#### 2.7.2. *In Vivo* Model

After three hours from the last oral gavage, FD-4 dissolved in saline (500 mg/kg body weight, 125 mg/mL) was orally administered to rats. Later, animals were euthanized, and serum levels were checked for FD-4 concentration after 3 hr using a fluorescence spectrophotometer (Perkin Elmer LS-55, USA) at an excitation wavelength of 485 nm and an emission wavelength of 530 nm [29].

### 2.8. Determination of Colonic Myeloperoxidase (MPO) Activity

Colonic tissue was extracted according to Bradley et al. [30] using hexadecyltrimethylammonium bromide (HTAB). The extracted sample was mixed with o-dianizidine HCl and hydrogen peroxide (H_2_O_2_), and absorbance was read spectrophotometrically at 460 nm. The sum of the MPO activity present in each sample/g tissue weight causes a change in spectrophotometric absorbance of 1/min at 460 nm [30].

### 2.9. Measurement of Serum Endotoxin Concentration

Serum endotoxin concentration levels were determined using the commercially available endotoxin quantitation kit (Thermo Fisher Scientific) following the kit manufacturer's guidelines. Endotoxin concentrations were expressed in endotoxin units/ml.

### 2.10. Analysis of Colon Histology by H&E Staining

Colon tissue sections fixed in formalin solution were processed in OCT medium. Five to 10 *μ*M colon sections were cut using a cryostat device. To distinguish the morphological changes among the differentially treated rats, paraffin-fixed colon sections were stained with hematoxylin-eosin (HE) solution. All the slide sections were observed by a single investigator who was blinded to the treatment status. All images captured represent at least five random areas per colon section per treatment group.

### 2.11. Estimation of ROS Production

The amount of ROS was measured following the method used by Heidari et al. in 2016 [31] with modification. Briefly, colon tissue was was homogenized in 1 : 10 *w*/*v* Tris-HCl buffer (pH 7.4, 40 mM). After homogenization, 100 *μ*l of colon homogenate was mixed with 1 ml of Tris-HCl buffer and incubated with 5 *μ*l of carboxy-H2-DCFDA [5(6)-carboxy-2',7'-dichlorofluorescein diacetate] in the dark with a final concentration of 10 *μ*M at 37°C for 1 hr. The samples' fluorescence intensity was measured at 485 nm excitation wavelength and 525 nm emission wavelength for measuring the total of ROS production using a fluorescence spectrophotometer (Perkin Elmer LS-55, USA) [31].

### 2.12. Estimation of Colonic Oxidative Stress

#### 2.12.1. Estimation of the Colonic MDA Content by TBA Method

To make 10% homogenate, colon tissues were taken and processed with ice-cold potassium chloride (KCl) solution with the final concentration of 1.15% (*w*/*v*). In the colon homogenate, concentrations of MDA were measured by the TBA method [32] using Cayman Chemical MDA estimation kit. The supernatant obtained was measured spectrophotometrically at the absorbance of 532 nm (UV-visible spectrophotometer model: PharmaSpec UV-1700, Shimadzu, Japan). For the calibration, each standard sample was repeated three times (*n* = 3). A blank sample was repeated (*n* = 5) replacing the standard or sample with a TCA-TBA-HCl reagent. Tissue protein levels were measured by the Lowry method, and the total MDA concentration obtained from each sample was then normalized to the protein concentration of the respective sample.

#### 2.12.2. Estimation of the Serum MDA Content by HPLC Method

500 *μ*l serum samples were mixed with 6 M sodium hydroxide (NaOH) (100 *μ*l) and incubated at 60°C in a water bath for 45 min. The hydrolyzed samples were mixed with 35% perchloric acid (250 *μ*l) for acidification and centrifuged for 10 min at 15000 × g. Later, supernatant (250 *μ*l) was mixed with 2,4-dinitrophenylhydrazine (DNPH) (25 *μ*l) solution and incubated in the dark for 10 min. The derivatized serum sample was then analyzed using HPLC apparatus (Waters Breeze-2, USA), through the ODS2 reverse-phase column. Acetonitrile and HPLC-grade water having 0.2% acetic acid at a ratio of 38 : 62 was used as mobile phase. HPLC was done under isocratic conditions with a flow rate of 1.0 ml/min, and MDA content in the samples was detected at 310 nm using a UV detector.

*(1) Preparation of Standard Curve*. 20 nmol/ml of MDA standard stock solution was prepared from 1,1,3,3-tetraethoxypropane (TEP) (TCI, Japan), and further diluted with 1% H_2_SO_4_ to yield a final concentration of 0.10, 0.20, 0.31, 0.62, 1.25, 2.50, 5.00, and 10.00 nM/ml of MDA. To 250 *μ*l of each standard sample, 25 *μ*l of DNPH was added and incubated for 10 min in the dark [33].

#### 2.12.3. Evaluation of Antioxidant Capacity

The colon tissue was mechanically homogenized and centrifuged at 3000 × g for 15 min. The colon tissue weight to physiological saline ratio was 1 : 9 *v*/*v*. Glutathione peroxidase (GSH-Px), superoxide dismutase (SOD), and catalase (CAT) in the supernatant of homogenate were detected by using commercial assay kits (Thermo Fisher Scientific, USA).

### 2.13. Estimation of Butyrate Levels in the Serum

20 *μ*l serum samples were mixed with 100 *μ*l methanol, followed by a vigorous vortex. Later, the mixture was centrifuged at 4800 × g for 10 min at 4°C, and further supernatant was collected and analyzed through the C18 chromatographic column. Acetonitrile and HPLC-grade water having 0.1% phosphoric acid solution at a ratio of 20 : 80 was used as a mobile phase. HPLC was done under isocratic conditions with a flow velocity of 1.0 ml/min, and butyrate levels in the sample were detected at 206 nm using a UV detector [34].

#### 2.13.1. Standard Curve Preparation

We diluted 0.1 g butyrate standard solution with HPLC-grade water to 100 ml and filtered it through a 0.45 *μ*m Millipore filter. Standard solution was introduced at 0 *μ*l, 5 *μ*l, 10 *μ*l, 20 *μ*l, 30 *μ*l, and 50 *μ*l.

### 2.14. Extraction of RNA and Quantitative Reverse Transcription Polymerase Chain Reaction (qRT-PCR)

Caco-2 monolayer cells were seeded in 6-well plates at the seeding density of 2.0 × 10^5^ cells/ml and cultured for 21 days. Afterward, probiotic V (10 *μ*l), Met (1 mM), and ethanol (100 mM) were added according to their respective groups and incubated for 48 h. Cells were harvested using the TRIzol Reagent extraction method. Also, for *in vivo* studies, a total of 4 mg of colonic RNA was isolated from the colon tissue using the TRIzol Reagent extraction method. The concentrations (ng/*μ*l) and purity (A260/A280) of extracted RNA were measured by a NanoDrop instrument (Thermo Fisher Scientific, Waltham, MA, USA). Extracted RNA was given a DNase treatment, and cDNA was synthesized from 1 *μ*g of total RNA using a first-strand cDNA synthesis kit, according to the manufacturer's protocol. Real-time PCR amplification for quantifying the gene expressions was done using the SYBR/ROX Master Mix in an Agilent Mx3005P qPCR system (Agilent Stratagene) for the following primers (Tables 1 and 2). The respective gene expressions were normalized to the 18S rRNA gene expression (endogenous control). The quantification of results was done using the 2^−ΔΔCT^ method [35] and expressed as fold-over basal change comparative to the control group.

### 2.15. Statistical Analysis

The values of the replicates were calculated as mean ± SD. To evaluate the statistical data, GraphPad Prism 7 software using one-way analysis of variance (ANOVA) was used. Statistical variations among the different experimental sets of groups were considered to be significant at a *p* value less than 0.05.

### 2.16. *In Silico* Analysis

#### 2.16.1. Preparation of Proteins Using Homology Modeling

Due to the unavailability of 3D structures for *Rattus norvegicus*, protein modeling of SLC5A8 and Nrf-2 was performed based on known crystal structures from the Protein Data Bank (PDB) [36] using homology modeling through the SWISS-MODEL server [37]. The amino acid FASTA sequence of these three proteins used to build the model was retrieved from UniProt [38] (Accession ID: D3Z9E5, O54968, and Q80Z39, respectively). Afterward, the model was generated based on the quality analysis of the predicted model as conducted and was evaluated on the basis of Qualitative Model Energy Analysis (QMEAN) [39] and Global Model Quality Estimation (GMQE) score. GMQE score ranges between zero and one, and the score for the modeled protein closer to 1 indicates increased structural reliability. Another parameter of QMEAN *Z*-scores closer to zero indicate good agreement between the model structure and experimental structures of similar size. Scores of -4.0 or below is an indication of models with low quality, highlighted by a change of the “thumbs-up” symbol to a “thumbs-down” symbol next to the score. Further, MolProbity 4.5.1 [40] was used to build the Ramachandran plot to analyze the psi and phi angles of the modeled proteins. Target-template alignment of sequences of the three proteins for their template protein sequences was aligned using ClustalW [41]. The crystal structures for *Rattus norvegicus* GPR109A were built using the Robetta server (http://robetta.bakerlab.org) [42], and the Ramachandran plot was built using PROCHECK [43] on SAVES server v6.0 (https://saves.mbi.ucla.edu/). The X-ray-diffracted 3D structure for *Rattus norvegicus* heme oxygenase (PDB ID: 6J7A) was available, and hence, directly retrieved from PDB.

#### 2.16.2. Molecular Docking

*In silico* experiments were performed to study the protein-ligand interaction and binding affinity [44]. For performing protein-ligand interaction, four of the proteins were first optimized for docking studies one by one and were prepared by assigning the hydrogen atoms and charges followed by energy minimization using the DockPrep tool [45]. Partial charges are assigned as an atom attribute as per the AM1-BCC method to generate high-quality atomic charges for protein. Charges for nonstandard residues, if any, were calculated using Amber's Antechamber module [46]. The energy minimization was performed using the 10,000 steepest descent steps with 0.02 Å step size and an update interval of 10 for all four proteins individually. All the steps mentioned above were performed using UCSF Chimera 1.15 [47].

The 3D conformations for both butyrate (CID: 104775) and Met (CID: 4091) to perform docking were retrieved from PubChem [48]. This was followed by optimization of both ligands through the Gasteiger algorithm [49] in the structure editing wizard of UCSF Chimera 1.15, which works on the chemoinformatic principle of electronegativity equilibration; the ligand files were saved in mol2 format, as per the methodology adopted previously in a published work [50, 51].

Out of four, all the three proteins Nrf-2, GPR109A, and SLC5A8 were modeled for species *Rattus norvegicus* and lacked the predefined ligand-binding site. Hence, the ligand-binding site information and the amino acids involved in the protein-ligand interaction at the cleft were chosen based on the crystallized ligand attached in the original PDB file of the template protein that was used to build the respective *Rattus norvegicus* protein structure. For HO-1 (PDB ID: 6J7A), the native ligand was removed before docking, and the protein was treated similarly to the modeled proteins. As we want to look into the synergistic effect of butyrate and Met working in tandem, it becomes important to screen all other potential ligand-binding sites as well on these four respective proteins. This was achieved by performing molecular protein-ligand docking in three stages: (i) Blind or global docking which enables identifying the ligand-binding sites on multiple cavities in and on the structure of a particular protein; this strategy is particularly advantageous when the ligand-binding sites on the structure of the proteins are unknown, like in the case of this study. (ii) Single Ligand Localized docking (SLLD) which enables identifying the actual orientation of the ligand bound to a protein at the binding site. (iii) Multiple Ligand Simultaneous Docking (MLSD), as the name suggests, allows simultaneous interactions of multiple ligands at the active cleft of the protein. MLSD varies from SLLD by allowing different conformations of multiple ligands at random to run simultaneously. For running MLSD, the individual docking parameter files for each ligand, i.e., butyrate and Met, were merged as one, minimized again, and saved as an AutoDock ligand in pdbqt format.

Protein-ligand docking analysis was conducted using AutoDock Vina [52], and the program has executed an add-on in Chimera. Of all the possible poses suggested after docking both the ligands with an individual protein, the pose showing maximum hydrogen bond-forming ability and minimum binding free energy change (kcal/mol) as observed in the ViewDock window were chosen as the best-docked pose. Best poses were then visualized in BIOVIA Discovery Studio [53] for hydrogen bond formation by the functional groups of ligands with amino acids as part of the protein. The visualizer also suggested other supporting hydrophobic interactions made by the ligands in the cavity of the protein.

## 3. Results

### 3.1. Combinatorial Treatment of Probiotic V and Met Improves the Increased Colon Weight to Length Ratio in the Rat Model of Ethanol-Induced Intestinal Barrier Injury

**Figure 1:**
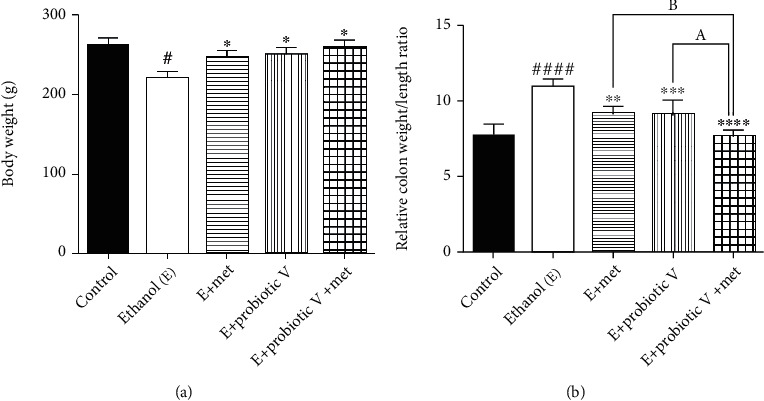
Effect of probiotic V and Met unaided or in combination on ethanol-induced gut dysbiosis. (a) Body weight. (b) Relative colon weight to length ratio. Values are expressed as mean ± SD of six rats. Statistical analysis: one-way ANOVA followed by Tukey's post hoc test. ^#^*p* < 0.05 and ^####^*p* < 0.0001 compared to the control group; ^∗^*p* < 0.05, ^∗∗^*p* < 0.01, ^∗∗∗^*p* < 0.001, and ^∗∗∗∗^*p* < 0.0001 compared with the ethanol-fed group; ^a^*p* < 0.05 compared with the E + probiotic V group; ^b^*p* < 0.05 compared with the E + Met group.

Figure 1 shows decreased body weight (BW) and increased colon weight/length ratio in the ethanol-fed group as compared to the control group. Interestingly, administration of probiotic V and Met in combination significantly increased the BW and decreased the colon weight/length ratios compared to the ethanol-fed groups and individual treatment of either probiotic V or Met. However, nonsignificant differences were observed in the case of BW when compared to the individual treatment of either probiotic V or Met.

### 3.2. Combinatorial Treatment of Probiotic V and Met Improves Histological Modification in Colon Tissue

**Figure 2:**
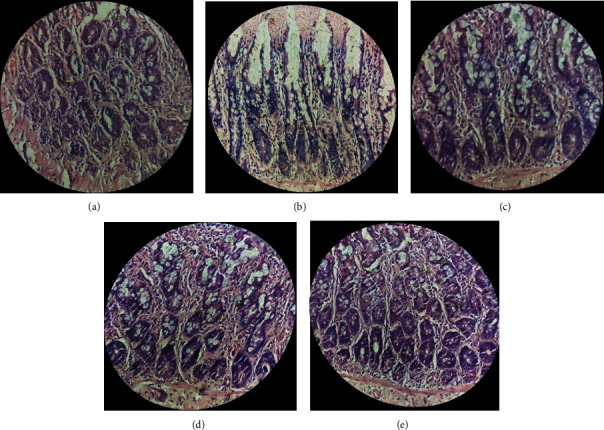
Effect of probiotic V and Met unaided or in combination on histopathological modifications in inhibiting the ethanol-induced intestinal injury. (a) Gross examination of rat colon morphology (400x magnification). Microscopic images are as follows: (a) Control rats supplemented with maltodextrin (substituted isocalorically). (b) Ethanol-fed rats supplemented with increasing concentrations of ethanol. (c) Met: ethanol-fed rats supplemented with 75 mg/kg Met. (d) Probiotic V: ethanol-fed rats supplemented with probiotic V (10^8^ CFU/day). (e) Probiotic V and Met: ethanol-fed rats supplemented with a mixture of probiotic V (10^8^ CFU/day) and Met (75 mg/kg).

Analysis of the HE-stained colon sections of control rats demonstrated the normal architecture of colon tissue with straight tubular glands lined by well-organized epithelial cells and lining as well as crypts of Lieberkühn. The vertically oriented crypts were lined by columnar epithelial cells. The ethanol-fed group showed disorganized epithelial cells, deformed crypts, and mucosal lining of colon. The surface area of crypts of Lieberkühn also showed remarkable reduction. However, histological analysis of the ethanol-fed group treated with the combined administration of probiotic V and Met showed histology similar to the normal group (Figure 2).

### 3.3. Combinatorial Treatment of Probiotic V and Met Attenuates Ethanol-Induced Disruption of TJ Expression and Intestinal Barrier Dysfunction

**Figure 3:**
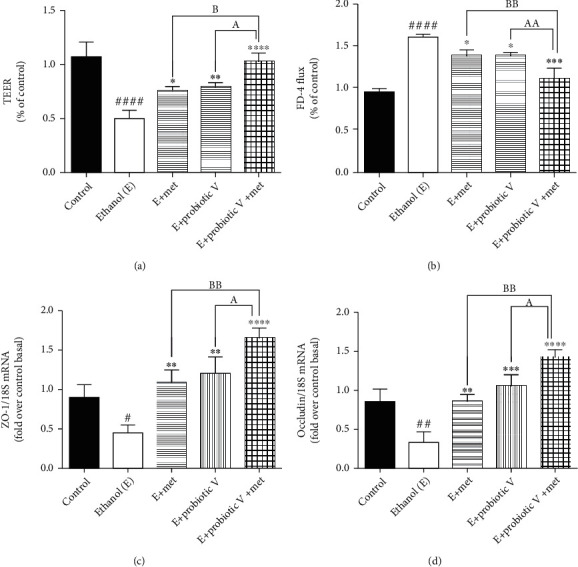
Effect of probiotic V and Met unaided or in combination on ethanol-induced intestinal barrier integrity and permeability in Caco-2 monolayers. (a) TEER. (b) FD-4 influx. Gene expression levels of (c) ZO-1 and (d) occludin. Values are expressed as mean ± SD of three individual experiments. Statistical analysis: one-way ANOVA followed by Tukey's post hoc test. ^#^*p* < 0.05, ^##^*p* < 0.01, and ^####^*p* < 0.0001 compared to the control group; ^∗^*p* < 0.05, ^∗∗^*p* < 0.01, ^∗∗∗^*p* < 0.001, and ^∗∗∗∗^*p* < 0.0001 compared with the ethanol-fed group; ^a^*p* < 0.05 and ^aa^*p* < 0.01 compared with the E + probiotic V group; ^b^*p* < 0.05 and ^bb^*p* < 0.01 compared with the E + Met group.

**Figure 4:**
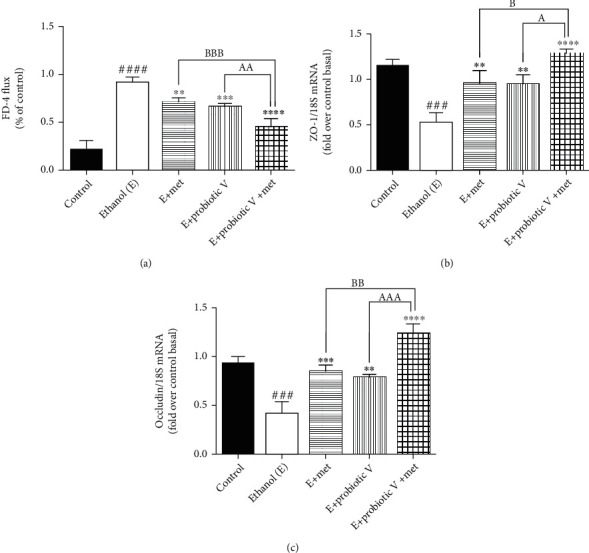
Effect of probiotic V and Met alone or in combination on ethanol-induced intestinal barrier integrity in the Wistar rat model. (a) Percentage of FD-4 permeation. TJ protein expression of (b) ZO-1 and (c) occludin. Values are expressed as mean ± SD of six rats. Statistical analysis: one-way ANOVA followed by Tukey's post hoc test. ^###^*p* < 0.001 and ^####^*p* < 0.0001 compared to the control group; ^∗∗^*p* < 0.01, ^∗∗∗^*p* < 0.001, and ^∗∗∗∗^*p* < 0.0001 compared with the ethanol-fed group; ^a^*p* < 0.05, ^aa^*p* < 0.01, and ^aaa^*p* < 0.001 compared with the E + probiotic V group; ^b^*p* < 0.05, ^bb^*p* < 0.01, and ^bbb^*p* < 0.001 compared with the E + Met group.

TJs exert a crucial role in preventing gut integrity. Concerning the gene expression analysis of TJ proteins including ZO-1 and occludin, the transcriptions of both genes were significantly reduced in ethanol-exposed Caco-2 monolayers (Figures 3(c) and 3(d)) as well as the colon (Figures 4(b) and 4(c)). The expression of ZO-1 and occludin demonstrated an overall trend of upregulation after the individual treatment of probiotic V or Met, which was further more significantly upregulated by the two in the combinations.

In parallel, we also determined the combinatorial effect of probiotic V and Met on intestinal epithelial cell integrity *in vitro* and *in vivo*. *In vitro*, results showed that cells exposed to ethanol showed a significant decrease in TEER measurement indicating ethanol markedly disrupts the intestinal epithelial barrier. However, the decreased TEER induced by ethanol was significantly improved by the combined treatment of probiotic V and Met when compared with the ethanol-fed group as well as probiotic V- and Met-unaided groups (Figure 3(a)). Correspondingly, the cells incubated with ethanol greatly increased the FD-4 permeation over that in the control group, which was significantly attenuated by the combined treatment of probiotic V and Met in comparison to the ethanol group as well as either the probiotic V- or Met- (*p* < 0.05) unaided groups (Figure 3(b)). In the *in vivo* model, mucosal permeability to FD-4 flux (Figure 4(a)) was higher in ethanol-fed rats compared to the control group. Ethanol feeding, however, failed to increase FD-4 flux permeability and endotoxins levels, when administered with probiotic V or Met individually. Moreover, combined administration of probiotic V and Met significantly decreased the FD-4 permeation compared to the individual treatment of probiotic V or Met. Taken together, the above results indicate that combined treatment of probiotic V and Met restored the damaged TJs, and intestinal barrier function caused by the ethanol in an *in-vitro* and *in-vivo* model of intestinal barrier injury.

### 3.4. Combinatorial Treatment of Probiotic V and Met Attenuates Ethanol-Induced Intestinal Oxidative Stress

**Figure 5:**
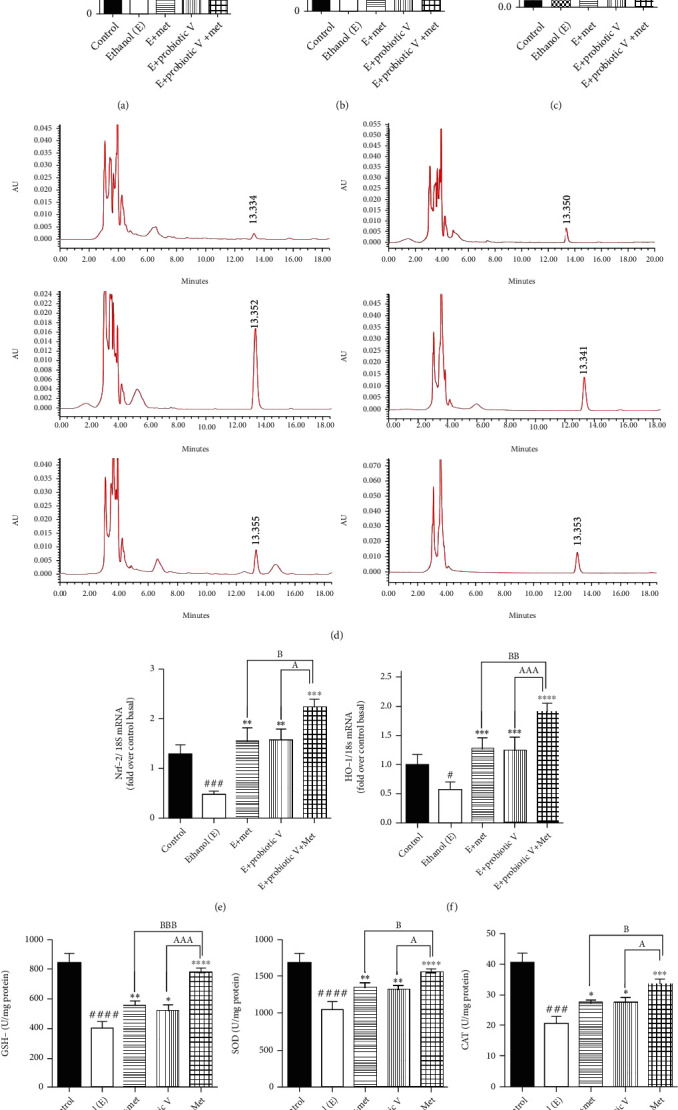
Probiotic V and Met unaided or in combination prevent ethanol-mediated ROS production and oxidative stress in ethanol-induced intestinal injury. (a) Fluorescence spectroscopy with excitation/emission wavelengths at 485 nm/525 nm after incubation with carboxy-H2-DCFDA. (b) Total serum MDA levels. Here, the MDA levels in serum were quantified with reference to the standard area under the curve (AUC). (c) Total MDA concentration in colon tissue. The MDA content was quantified using the TBA assay method. (d) HPLC chromatograms of total serum MDA after DNPH derivatization in (a) standard, (b) control, (c) ethanol-fed, (d) Met (75 mg/kg) + ethanol-fed, (e) probiotic V (10^8^ CFU/day) + ethanol-fed, and (f) probiotic V (10^8^ CFU/day) + Met (75 mg/kg) + ethanol-fed rats. The amount of MDA present in serum was estimated after processing and DNPH derivatization. The serum sample after derivatization was then analyzed by a Shimadzu HPLC instrument using a fully end-capped spherical ODS2 C18 reverse-phase HPLC column. For the mobile phase, acetonitrile-distilled water with a ratio of 38 : 62 consisting of 0.2% glacial acetic acid was used. Colonic expression of antioxidants (e) Nrf-2, (f) HO-1, (g) GSH-Px, (h) SOD, and (i) CAT. The gene expression levels were measured after normalizing against 18S. Values are expressed as mean ± SD of six rats. Statistical analysis: one-way ANOVA followed by Tukey's post hoc test. ^#^*p* < 0.05, ^###^*p* < 0.001, and ^####^*p* < 0.0001 compared to the control group; ^∗^*p* < 0.05, ^∗∗^*p* < 0.01, ^∗∗∗^*p* < 0.001, and ^∗∗∗∗^*p* < 0.0001 compared with the ethanol-fed group; ^a^*p* < 0.05, ^aaa^*p* < 0.001, and ^aaaa^*p* < 0.0001 compared with the E + probiotic V group; ^b^*p* < 0.05, ^bb^*p* < 0.01, and ^bbb^*p* < 0.001 compared with the E + Met group.

To explore the combined effect of probiotic V and Met on intestinal oxidative stress, we investigated the ROS generation on rat colon tissues. We observed that the production of ROS was promoted manifestly by ethanol stimulation; meanwhile, either individual treatment of probiotic V or Met noticeably reduced the ROS accumulation, which was further dramatically diminished by the two in combination as compared to the ethanol group as well as individual treatment of probiotic V or Met (Figure 5(a)). Increased ROS production caused lipid peroxidation, thus resulting in malondialdehyde (MDA) generation.

Using HPLC, serum analysis of MDA levels showed higher MDA content in the ethanol-fed group (83.48 ± 0.89 nM/ml) compared to the control group (32.33 ± 0.82 nM/ml), indicating that severe oxidative stress occurred due to ethanol. On the other hand, in the group with combinatorial treatment of probiotic V and Met cotreated with ethanol, the MDA content decreased to normal levels (37.08 ± 0.72 nM/ml) compared to the ethanol-fed group as well as with either individual agent probiotic V (59.83 ± 3.78 nM/ml) or Met (67.40 ± 1.94 nM/ml). The colonic MDA content in the rats was measured by the TBA method. The MDA concentration in ethanol-fed rats was found to be 1.07 ± 0.06 *μ*M/ml as compared to the control group (0.06 ± 0.01 *μ*M/ml). However, feeding the rats with probiotic V and Met in combination along with the ethanol showed reduced MDA levels to 0.22 ± 0.02 *μ*M/ml compared with the ethanol-fed group as well as with either individual agent probiotic V (0.46 ± 0.04 *μ*M/ml) or Met (0.47 ± 0.05 *μ*M/ml) (Figures 5(b)–5(d)).

Under oxidative stress, transcription factor-like nuclear factor-like 2 (Nrf2) and enzyme-like heme-oxygenase (HO-1) plays a role in the regulation of antioxidant machinery. Ethanol feeding caused a graphic reduction in expression levels for the Nrf-2 and HO-1 genes in colonic mucosa. The combined administration of probiotic V and Met significantly increased the expression levels of Nrf-2 and HO-1 compared to the ethanol-fed groups as well as the probiotic V or Met-unaided groups (Figures 5(e) and 5(f)). The treatment with either probiotic V or Met alone upregulated the activities of GSH-Px, SOD, and CAT which was further elevated by the combined treatment of probiotic V and Met of the ethanol-fed group as well as individual treatment of either probiotic V or Met (Figures 5(g)–5(i)).

**Figure 6:**
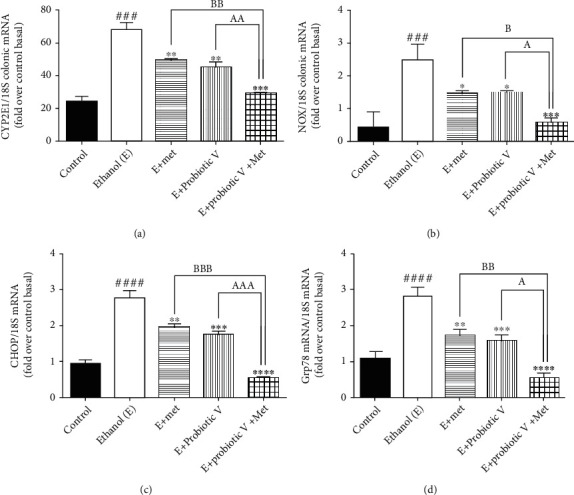
Probiotic V and Met alone or in combination prevents ethanol-mediated oxidative stress and endoplasmic reticulum (ER) stress in the male Wistar rat model. mRNA expression of (a) CYP2E1, (b) NOX and ER stress gene (c) CHOP, and (d) Grp78 in the male Wistar rat colon. The gene expression levels were measured after normalizing against 18S. Values are expressed as mean ± SD of six rats. Statistical analysis: one-way ANOVA followed by Tukey's post hoc test. ^###^*p* < 0.001 and ^####^*p* < 0.0001 compared to the control group; ^∗^*p* < 0.05, ^∗∗^*p* < 0.01, ^∗∗∗^*p* < 0.001, and ^∗∗∗∗^*p* < 0.0001 compared with the ethanol-fed group; ^a^*p* < 0.05, ^aa^*p* < 0.01, and ^aaa^*p* < 0.001 compared with the E + probiotic V group; ^b^*p* < 0.05, ^bb^*p* < 0.01, and ^bbb^*p* < 0.001 compared with the E + Met group.

**Figure 7:**
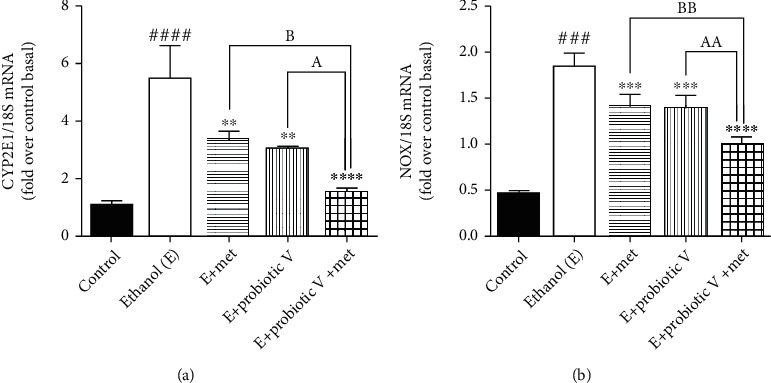
Probiotic V and Met alone or in combination prevents ethanol-mediated oxidative stress in Caco-2 monolayer cells. mRNA expression of (a) CYP2E1 and (b) NOX. The gene expression levels were measured after normalizing against 18S. Values are expressed as mean ± SD of three individual experiments. Statistical analysis: one-way ANOVA followed by Tukey's post hoc test. ^###^*p* < 0.001 and ^####^*p* < 0.0001 compared to the control group. ^∗∗^*p* < 0.01, ^∗∗∗^*p* < 0.001, and ^∗∗∗∗^*p* < 0.0001 compared with the ethanol-fed group; ^a^*p* < 0.05 and ^aa^*p* < 0.01 compared with the E + probiotic V group; ^b^*p* < 0.05 and ^bb^*p* < 0.01 compared with the E + Met group.

Ethanol metabolism involving CYP2E1 enzyme and NADPH-oxidase (NOX) causes lipid peroxidation, and further, it forms protein adducts as a result of the end product of lipid peroxidation. This also affects the ER functioning and protein folding. *In vivo*, levels of ER stress markers like CHOP and Grp78 were significantly elevated in the ethanol-fed group as compared to control group (Figures 6(c) and 6(d)). Also, *in vitro* and *in vivo*, levels of CYP2E1 enzyme and NOX were significantly elevated in the ethanol group in comparison to the control group. The treatment with either probiotic V or Met alone downregulated the activities of CYP2E1, NOX (Figures 6(a), 6(b), and 7), CHOP, and Grp78, which were further reduced by the combined treatment of probiotic V and Met of the ethanol-fed group as well as individual treatment of either probiotic V or Met. Our study demonstrated that probiotic V and Met in the presence of ethanol is effective in preventing the ethanol-induced oxidative stress along with ER stress in both cells and colon tissue of rat model of gut injury.

### 3.5. Combinatorial Treatment of Probiotic V and Met Blocks Ethanol-Induced Intestinal Inflammation

**Figure 8:**
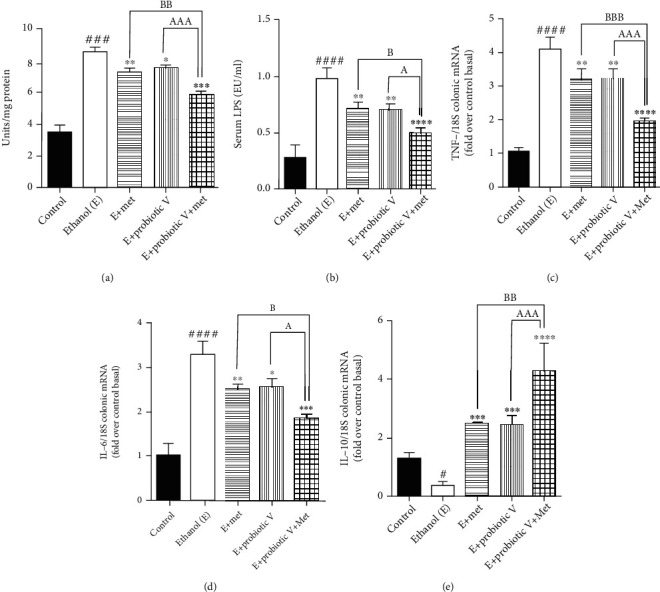
Probiotic V and Met alone or in combination inhibits ethanol-induced intestinal inflammation in the male Wistar rat model. (a) Effect on the myeloperoxidase activities in the colon. (b) Effect on the LPS levels in the blood. The inflammatory gene levels in the male Wistar rat colon of (c) TNF-*α* and (d) IL-6 and anti-inflammatory gene level of (e) IL-10. The gene expression levels were measured after normalizing against 18S. Values are expressed as mean ± SD of six rats. Statistical analysis: one-way ANOVA followed by Tukey's post hoc test. ^#^*p* < 0.05, ^###^*p* < 0.001, and ^####^*p* < 0.0001 compared to the control group; ^∗^*p* < 0.05, ^∗∗^*p* < 0.01, ^∗∗∗^*p* < 0.001, and ^∗∗∗∗^*p* < 0.0001 compared with the ethanol-fed group; ^a^*p* < 0.05 and ^aaa^*p* < 0.001 compared with the E + probiotic V group; ^b^*p* < 0.05, ^bb^*p* < 0.01, and ^bbb^*p* < 0.001 compared with the E + Met group.

There is a strong piece of evidence demonstrating the link between intestinal barrier dysfunction and intestinal inflammation. Myeloperoxidase (MPO) is considered the hallmark of inflammation in the tissue. Also, endotoxin, i.e., serum LPS levels are known to be important to determine the translocation of bacterial products and intestinal permeability. In the current study, we observed that colonic MPO activity and serum LPS levels were significantly upregulated in ethanol-fed rats compared to control rats. Ethanol-fed rat co-treated with either probiotic V or Met showed reduced colonic MPO activity (Figure 8(a)) and serum LPS levels (Figure 8(b)) compared to the ethanol groups, which were more significantly reduced by the combinatorial treatment of probiotic V and Met.

**Figure 9:**
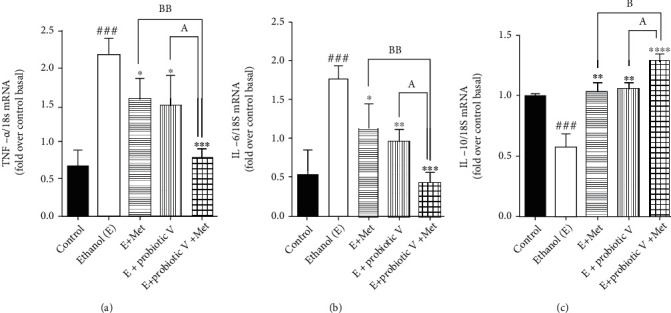
Probiotic V and Met alone or in combination inhibits ethanol-induced intestinal inflammation in the Caco-2 monolayers. Inflammatory gene levels of (a) TNF-*α* and (b) IL-6 and anti-inflammatory gene level of (c) IL-10. The gene expression levels were measured after normalizing against 18S. Values are expressed as mean ± SD of three individual experiments. Statistical analysis: one-way ANOVA followed by Tukey's post hoc test. ^###^*p* < 0.001 compared to the control group; ^∗^*p* < 0.05, ^∗∗^*p* < 0.01, ^∗∗∗^*p* < 0.001, and ^∗∗∗∗^*p* < 0.0001 compared with the ethanol-fed group; ^a^*p* < 0.05 compared with the E + probiotic V group; ^b^*p* < 0.05 and ^bb^*p* < 0.01 compared with the E + Met group.

Similarly, by analyzing the gene expression by qRT-PCR, we found that the ethanol group showed an elevation in the expression levels of proinflammatory genes like TNF-*α* and IL-6, compared to the control group. Downregulation of anti-inflammatory expression levels of IL-10 was observed in the ethanol group. Treatment with either probiotic V or Met fundamentally switched all the expression levels, i.e., decreased expression levels of TNF-*α* and IL-6, as well as increased expression levels of IL-10, when compared with the ethanol group, which was equally improved by the combinatorial treatment when compared with either individual treatment of probiotic V or Met (Figures 8(c)–8(e) and 9). The results suggest that combined treatment of probiotic V and Met can prevent ethanol-induced intestinal inflammation by regulating the levels of TNF-*α* and IL-6 in an *in vitro* and *in vivo* model of intestinal barrier injury.

### 3.6. Combinatorial Treatment of Probiotic V and Met Regulates the Lipid Metabolism in the Colonic Mucosa of the Rat Model

**Figure 10:**
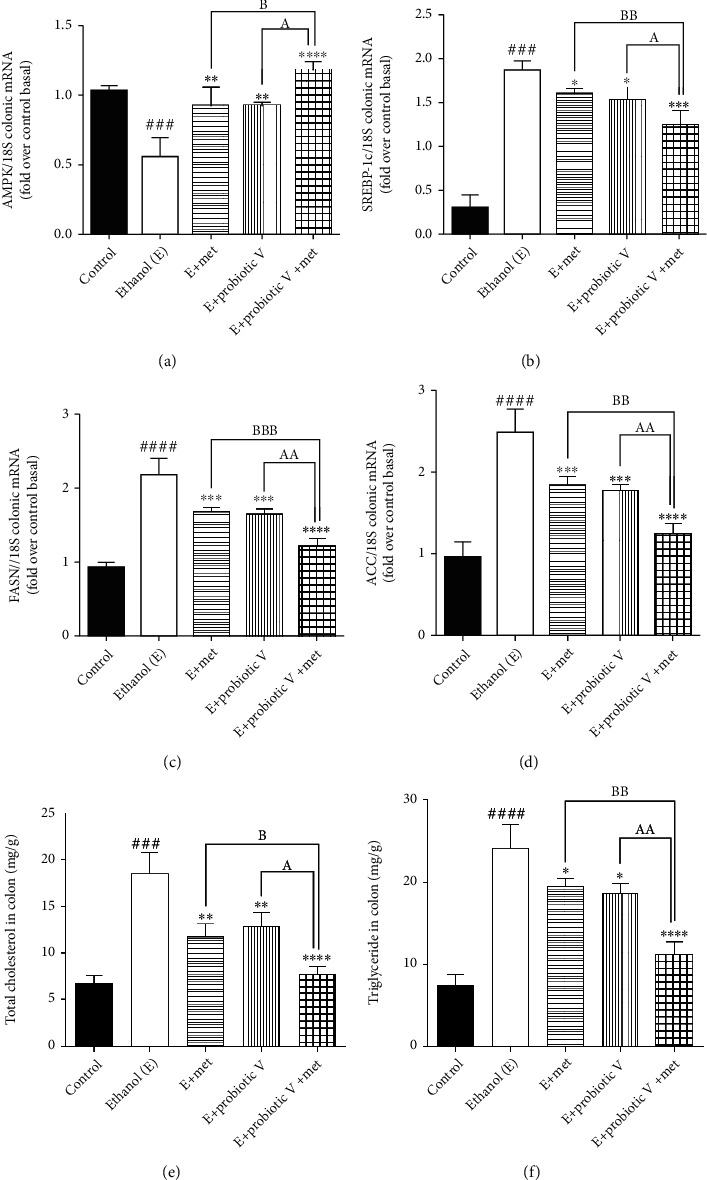
Probiotic V and Met unaided or in combination regulate the lipid accumulation in the colon to prevent ethanol-induced intestinal injury. mRNA expression of lipogenic genes (a) AMPK, (b) SREBP-1c, (c) FAS, and (d) ACC involved in lipid metabolism in the rat colon and (e) levels of TC and (f) TG in the colon. The gene expression levels were measured after normalizing against 18S. Values are expressed as mean ± SD of six rats. Statistical analysis: one-way ANOVA followed by Tukey's post hoc test. ^###^*p* < 0.001 and ^####^*p* < 0.0001 compared to the control group; ^∗^*p* < 0.05, ^∗∗^*p* < 0.01, ^∗∗∗^*p* < 0.001, and ^∗∗∗∗^*p* < 0.0001 compared with the ethanol-fed group; ^a^*p* < 0.05 and ^aa^*p* < 0.01 compared with the E + probiotic V group; ^b^*p* < 0.05, ^bb^*p* < 0.01, and ^bbb^*p* < 0.001 compared with the E + Met group.

To determine the combined effect of probiotic V and Met in protecting the intestinal barrier, we determined the adenosine monophosphate-activated protein kinase (AMPK) levels. Indeed, chronic alcohol consumption leads to abnormal lipid metabolism in the colonic mucosa by decreased AMPK activation which leads to increased sterol regulatory element-binding protein 1c (SREBP-1c) (also a key regulator of lipid metabolism) expression, further remarkably increasing lipogenesis by activating the downstream lipogenic genes, i.e., acetyl-CoA carboxylase (ACC) and fatty acid synthase (FAS) in ethanol-fed rats. Probiotic V or Met-unaided treatment upregulated the AMPK levels and lipid metabolism regulator, which is another way of being inhibited in the presence of ethanol. AMPK activation inhibits the expression of transcription factor, i.e., SREBP-1c, thereby preventing ethanol-induced lipogenesis. Consistent with the changed expression of SREBP-1c, the combination of probiotic V and Met also showed reduced expression levels of ACC and FAS as compared to the ethanol group, which was remarkably reduced as compared to individual treatment of probiotic V or Met (Figures 10(a)–10(d)).

Also, colonic contents of total cholesterol (TC) and triglyceride (TG) in the ethanol-fed rats, were significantly upregulated compared to those in the control group, which were significantly reduced in the combinatorial treatment of probiotic V and Met in comparison to the ethanol-fed group as well as the probiotic V or Met-unaided group (Figures 10(e) and 10(f)). The above results depicted that the combined treatment of probiotic V and Met could restore the colonic metabolic function damaged by ethanol.

### 3.7. Combinatorial Treatment of Probiotic V and Met Ameliorates the Butyrate Sensing against Ethanol Exposure

**Figure 11:**
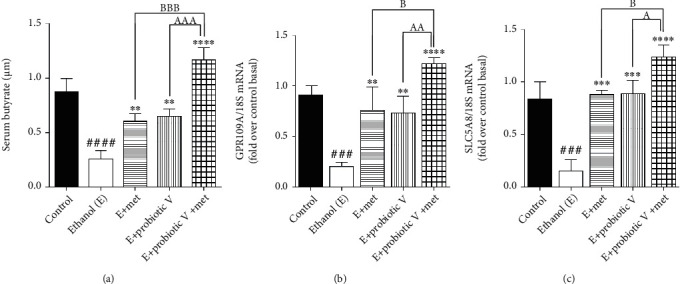
Probiotic V and Met unaided or in combination in enhancing the expression of butyrate receptor and transporter proteins in a rat model of ethanol-induced intestinal injury. Expression levels of (a) serum butyrate levels; (b) butyrate receptor: GPR109A; and (c) butyrate transporter: SLC5A8. The gene expression levels were measured after normalizing against 18S. Values are expressed as mean ± SD of six rats. Statistical analysis: one-way ANOVA followed by Tukey's post hoc test. ^###^*p* < 0.001 and ^####^*p* < 0.0001 compared to the control group; ^∗∗^*p* < 0.01, ^∗∗∗^*p* < 0.001, and ^∗∗∗∗^*p* < 0.0001 compared with the ethanol-fed group; ^a^*p* < 0.05, ^aa^*p* < 0.01, and ^aaa^*p* < 0.001 compared with the E + probiotic V group; ^b^*p* < 0.05 and ^bbb^*p* < 0.001 compared with the E + Met group.

The recent review literature provides the piece of evidence depicting increased levels of butyrate in the mice treated with probiotic VSL#3. The individual, as well as combinatorial treatment of probiotic V and Met, showed increased butyrate abundance in the *in vivo* model of ethanol-induced intestinal injury (Figure 11(a)). Taken together, also the expression of the butyrate receptor, i.e., GPR109A, and the butyrate transporter, i.e., SLC5A8, were reduced in the colon following the ethanol administration compared to the control group. In contrast, rats cotreated with either probiotic V or Met showed upregulated expression levels of GPR109A and SLC5A8 compared to the ethanol group (Figures 11(b) and 11(c)). Moreover, combined treatment of probiotic V and Met further improved more significantly, proving the prevention of intestinal barrier injury-induced inflammation.

### 3.8. Homology Modeling

The 3D model structures of *Rattus norvegicus* Nrf-2, GPR109A, and SLC5A8 were built using SWISS-MODEL based on their FASTA sequences. The model for *Rattus norvegicus* Nrf-2 protein was built using template protein PDB ID: 2DYH, X-ray diffraction “Structure of apelin receptor in complex with agonist peptide” of *Homo sapiens* with a resolution of 2.60 Å. This template was chosen as the best available template to build the 3D structure for *Rattus norvegicus* Nrf-2 considering the 22.57% sequence identity, 0.46 GMQE score generated by the target-template alignment of the modeled protein, and QMEAN score of -4.86. Molprobity results of the Ramachandran plot indicated 96.64% favored residues with only 0.34% amino acid residues marked as outliers, suggesting a satisfactory model (Supplementary Figures 1 and 2). For *Rattus norvegicus* GPR109A protein, template protein was built using the Robetta server and the results of Ramachandran plot were obtained using PROCHECK which indicated 92.3% favored residues with only 7.7% residues in a generously allowed region and 0.0% residues in disallowed regions (Supplementary Figures 3, 4, and 5). All these markers help us to predict that the proposed models are ideal modeled proteins for docking. Similarly, the model for *Rattus norvegicus* SLC5A8 was built using template protein PDB ID: 5NVA, an X-ray diffraction structure of “Substrate-bound outward-open state of a Na^+^-coupled sialic acid symporter reveals a novel Na^+^-site” of organism *Proteus mirabilis* HI4320 with a resolution of 2.26 Å. This template was chosen as the best available template to build the 3D structure for *Rattus norvegicus* SLC5A8 considering the 25.28% sequence identity, 0.52 GMQE score generated by the target-template alignment of the modeled protein, and QMEAN score of -5.94. The results for sequence identity and QMEAN *Z*-score indicate a low model quality. However, the Molprobity results of the Ramachandran plot indicated 95.97% favored residues with only 1.57% amino acid residues marked as outliers (Supplementary Figures 6, 7, and 8). Therefore, this proposed model was considered as an ideal modeled protein for *Rattus norvegicus* SLC5A8 and was chosen for molecular docking.

### 3.9. Molecular Docking

**Figure 12:**
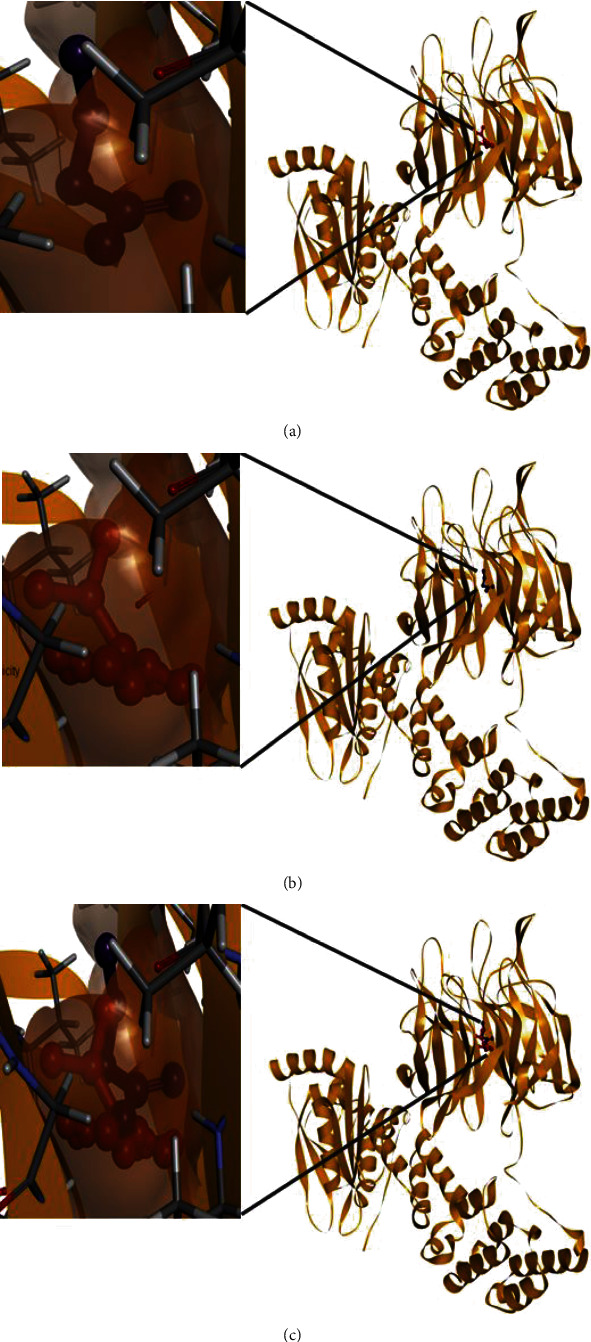
Molecular 3D interaction of *Rattus norvegicus* Nrf-2 (modeled using PDB ID: 2DYH) with (a) Met, (b) butyrate performed through SLLD, and (c) Met and butyrate together performed through MLSD.

**Figure 13:**
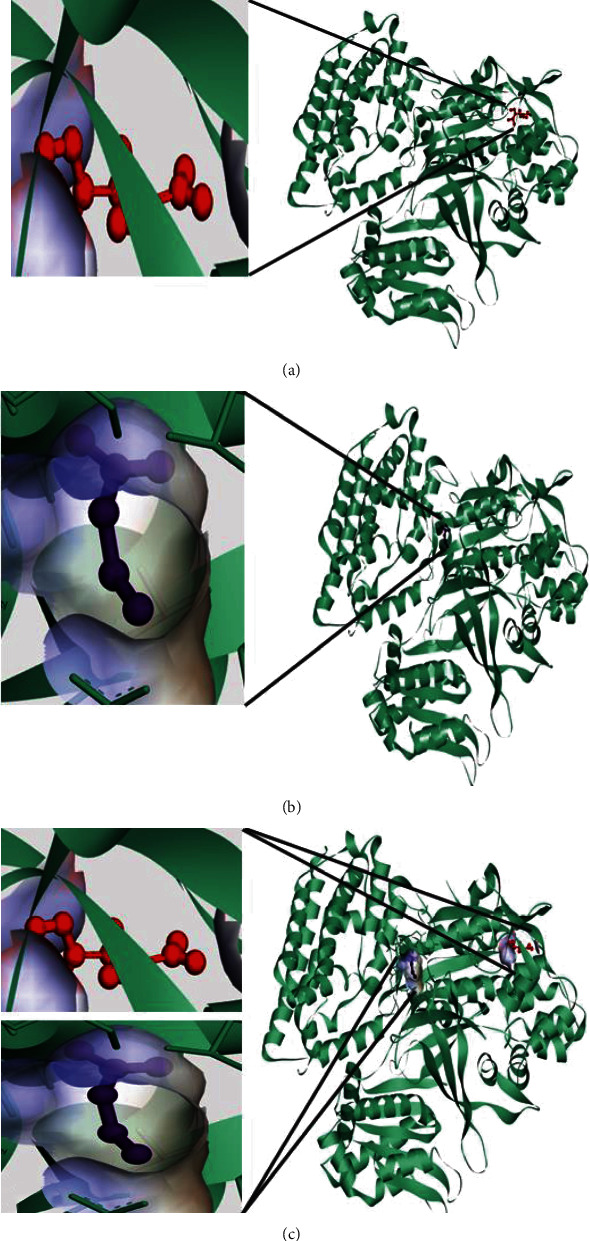
Molecular 3D interaction of *Rattus norvegicus* HO-1 (modeled using PDB ID: 6J7A) with (a) Met, (b) butyrate performed through SLLD, and (c) Met and butyrate together performed through MLSD.

**Figure 14:**
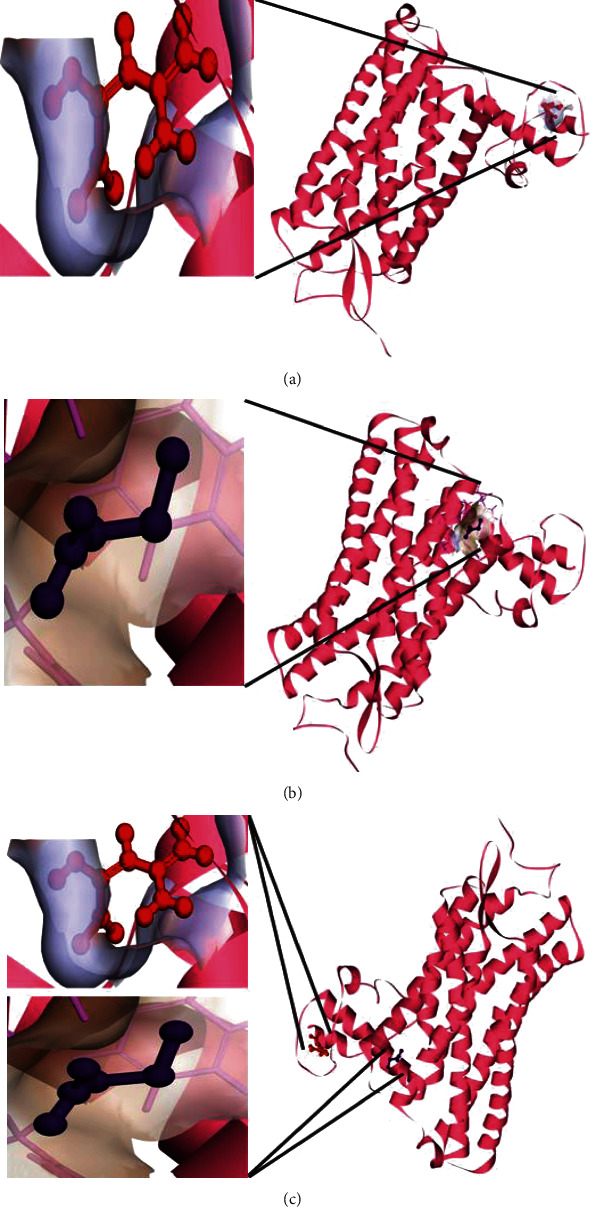
Molecular 3D interaction of *Rattus norvegicus* GPR109A (modeled using PDB ID: 5VBL) with (a) Met, (b) butyrate performed through SLLD, and (c) Met and butyrate together performed through MLSD.

**Figure 15:**
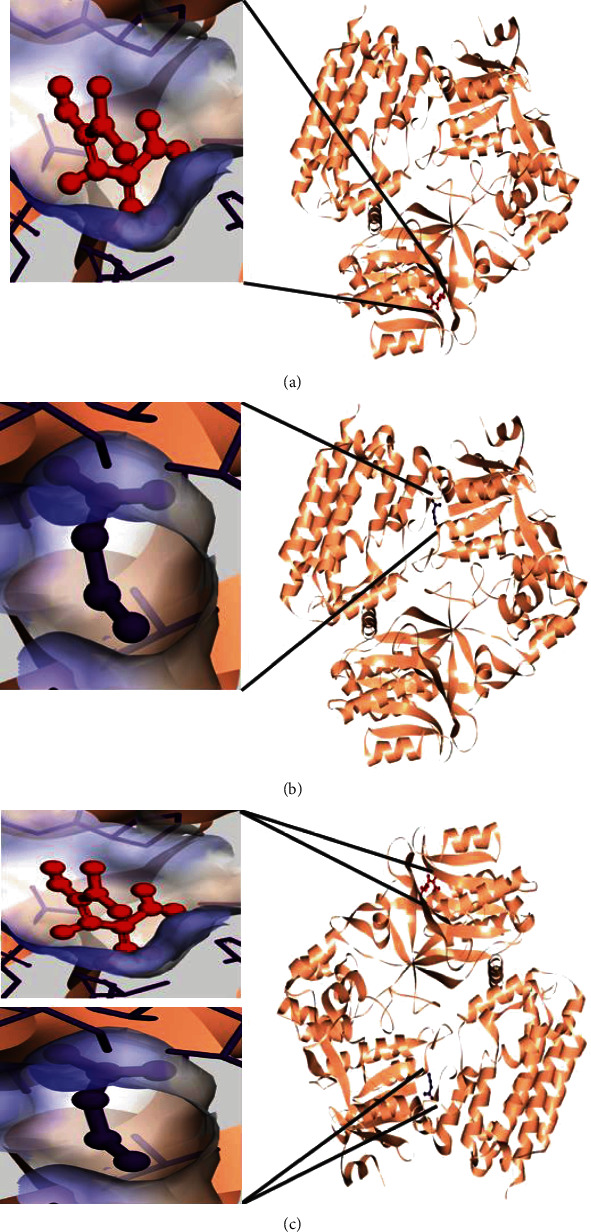
Molecular 3D interaction of *Rattus norvegicus* SLC5A8 (modeled using PDB ID: 5NVA) with (a) Met, (b) butyrate performed through SLLD, and (c) Met and butyrate together performed through MLSD.

**Figure 16:**
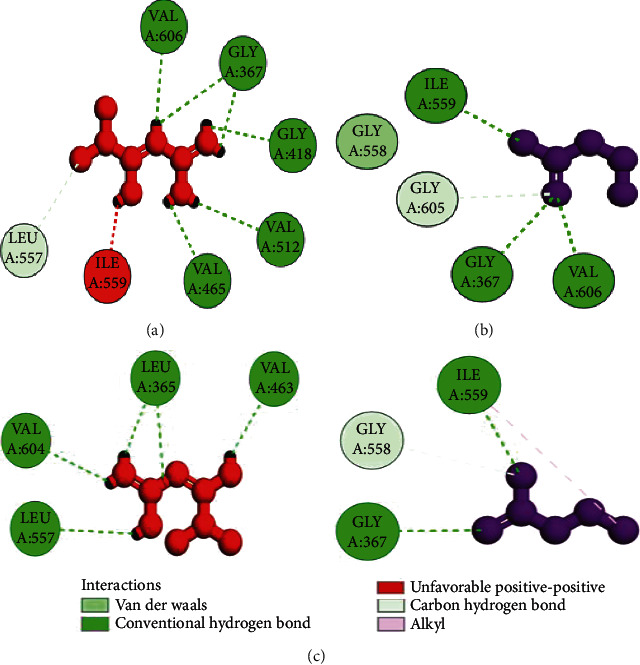
Molecular 2D interaction of *Rattus norvegicus* Nrf-2 (modeled using PDB ID: 2DYH) with (a) Met, (b) butyrate performed through SLLD, and (c) Met and butyrate together performed through MLSD.

**Figure 17:**
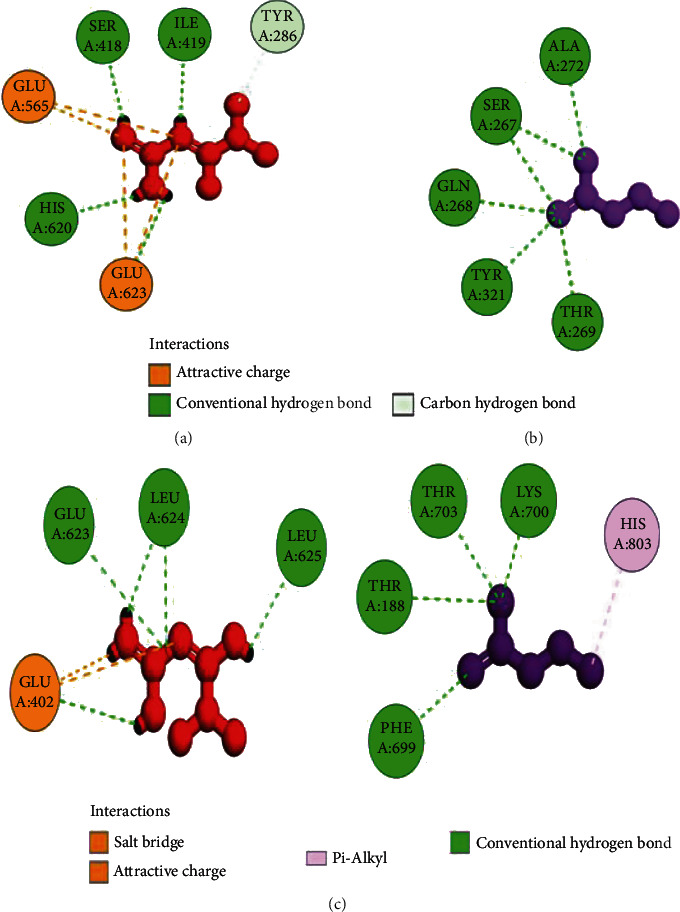
Molecular 2D interaction of *Rattus norvegicus* HO-1 (modeled using PDB ID: 6J7A) with (a) Met, (b) butyrate performed through SLLD, and (c) Met and butyrate together performed through MLSD.

**Figure 18:**
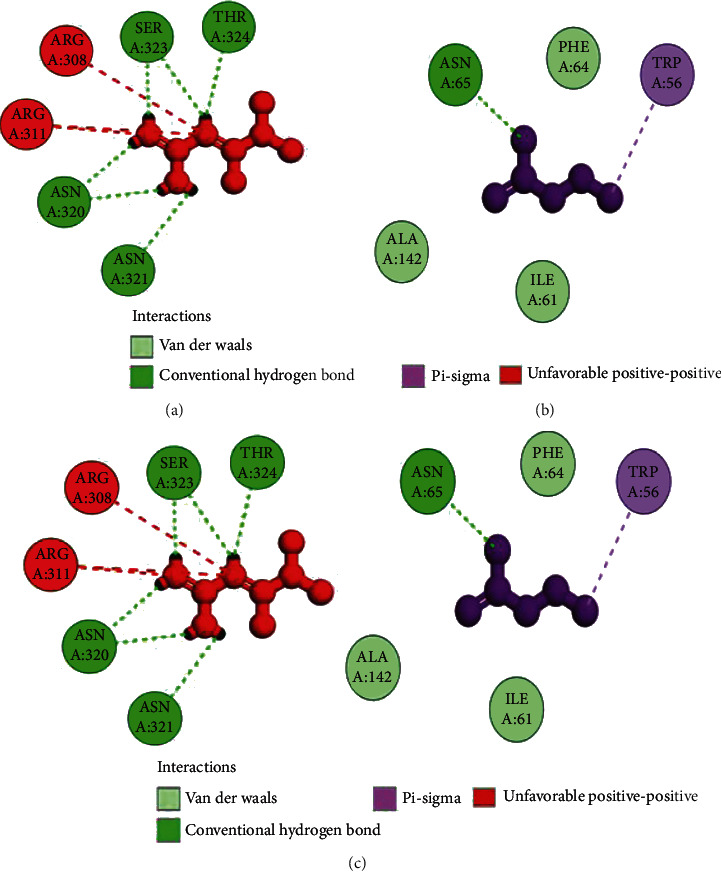
Molecular 2D interaction of *Rattus norvegicus* GPR109A (modeled using PDB ID: 5VBL) with (a) Met, (b) butyrate performed through SLLD, and (c) Met and butyrate together performed through MLSD.

**Figure 19:**
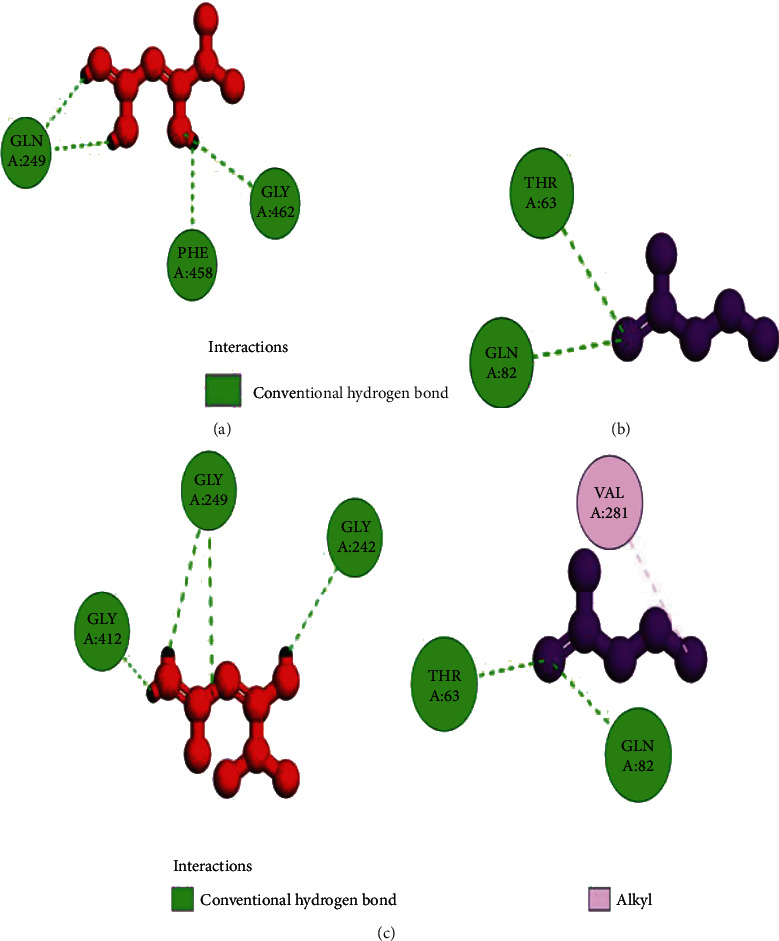
Molecular 2D interaction of *Rattus norvegicus* SLC5A8 (modeled using PDB ID: 5NVA) with (a) Met, (b) butyrate performed through SLLD, and (c) Met and butyrate together performed through MLSD.

The coordinates of the ligand-binding site on four of the proteins were determined using blind/global docking. With no prior ligand interaction profile, grid maps were prepared by fixing the grid box on the entire structure of the protein. Optimal pose within conformation of the target protein–ligand-binding sites were noted for both Met and butyrate on each of the proteins. It is to be noted that blind docking is more demanding in terms of computational time and the results are less accurate but help to predict the affinity of the ligand candidates in their optimal pose within the target protein. The single ligand localized docking strategy was then implemented to identify the exact orientation of the ligand on the protein and to validate the accuracy of the optimized pose in the identified binding cleft. As seen in Table 3, the Robetta-modeled Nrf-2 interaction with Met had a binding affinity of -6.0 kcal/mol, involving residues ILE: 559, VAL: 606, GLY: 367, GLY: 418, VAL: 512, and VAL: 465; with butyrate, it had a binding affinity of -3.4 kcal/mol, involving residues GLY: 605, ILE: 559, GLY: 367, and VAL: 606 (Figures 12(a) and 12(b)). HO-1 interaction with Met had a binding affinity of -5.7 kcal/mol, involving residues TYR: 286, ILE: 419, SER: 418, GLU: 565, HIS: 620, and GLU: 623; with butyrate, it had a binding affinity of -4.7 kcal/mol, involving residues ALA: 272, SER: 267, GLN: 268, TYR: 321, and THR: 269 (Figures 13(a) and 13(b)). The modeled GPR109A interaction with Met had a binding affinity of -4.9 kcal/mol, involving residues ASN: 321, ASN: 320, ARG: 308, SER: 323, THR: 324, and ARG: 311; with butyrate, it had a binding affinity of -3.4 kcal/mol, involving residues ILE: 162, ALA: 142, ASN: 65, PHE: 64, TRP: 56 (Figures 14(a) and 14(b)). Lastly, modeled SLC5A8 interaction with metformin had a binding affinity of -5.1 kcal/mol, involving residues GLN: 249, PHE: 458, and GLY: 462; with butyrate, it had a binding affinity of -4.7 kcal/mol, involving residues GLN: 82, and THR: 63 (Figures 15(a) and 15(b)). The results for MLSD reveal that Nrf-2 interaction with Met and butyrate had a simultaneous binding affinity of -9.7 kcal/mol, involving residues ASN: 321, VAL: 463, VAL: 604 LEU: 365, and LEU: 557 with Met, and ILE: 559, GLY: 367, and GLY: 558 with butyrate (Figure 12(c)). HO-1 interaction with Met and butyrate had a simultaneous binding affinity of -8.9 kcal/mol, involving residues GLU: 402, GLU: 623, LEU: 624, and LEU: 625 with Met, and PHE: 699, THR: 188, THR: 703, LYS: 700, and HIS: 803 with butyrate (Figure 13(c)). GPR109A interaction with Met and butyrate had a simultaneous binding affinity of -9.7 kcal/mol, involving residues ASN: 321, ASN: 320, ARG: 308, SER: 323, THR: 324, and ARG: 311 with Met, and ILE: 162, ALA: 142, ASN: 65, PHE: 64, and TRP: 56 with butyrate (Figure 14(c)). SLC5A8 interaction with Met and butyrate had a simultaneous binding affinity of -8.3 kcal/mol, involving residues GLN: 249, GLY: 412, and GLY: 242 with metformin, and GLN: 82, THR: 63, and VAL: 281 with butyrate (Figure 15(c)). Docking conformations were evaluated using these precalculated grid maps to enhance the robustness of docking. Binding energies and amino acid interaction for both the ligands are described in Table 3. There were several hydrophobic interactions such as alkyl and pi-alkyl bond formation occurring in the hydrophobic active site that is described in Figures 16, 17, 18, and 19.

## 4. Discussion

The effect of alcohol at the pathophysiological level contributes to a leaky gut or dysbiosis leading to intestinal hyperpermeability of intestinal bacteria [7, 8, 54]. Understanding the pathophysiological mechanism behind the ethanol-induced intestinal barrier dysfunction, as the present study executed, is considered to be significant with regard to biological and clinical manifestationbecause disruption of intestinal barrier integrity, called a leaky gut syndrome, is reflected to be one of the chief mechanisms involved in alcohol-mediated endotoxemia in the progression of ALD. Despite extensive research over the past decade, the molecular mechanisms for ethanol-induced intestinal barrier integrity dysfunction have remained unsuccessfully understood. During acute or chronic alcohol consumption, colon tissue has consistently been targeted.

At present, there are no available FDA-approved treatments to halt or cure alcoholic liver injury. Abstinence from drinking alcohol is known to be the only way to limit ALD development and progression. At this time, available treatments are single, and the ameliorative effect is not ideal [55]. One such individual known therapy is probiotics which protect against alcohol-induced gut leakiness [20]. For the first time, the current study proved the protective effect of Met against alcohol-induced gut leakiness. However, combined treatment of effective therapies can provide different links and targets to treat or prevent ethanol-induced gut dysbiosis through modulating intestinal mucosal barrier integrity, intestinal epithelial permeability, gut oxidation, and inflammatory response. Therefore, the current study cogitated that probiotic V in combination with Met could remarkably prevent the development of ethanol-induced intestinal barrier injury.

Recent studies demonstrated that chronic alcohol drinking leads to intestinal barrier dysfunction, increased intestinal permeability, and endotoxemia, which are considered to be initial events of ALD progression [56, 57]. The entire permeability of colon and Caco-2 monolayers were measured in the current study investigating the potential protective effect of combined treatment of probiotic V and Met on alcohol-induced intestinal mucosal barrier injury in both *in vitro* and *in vivo* models. Our results are following numerous studies, where Caco-2 monolayers and rat colonic epithelium presented decreased TEER (%) and increased colonic mucosal permeability to FITC-inulin was observed in the ethanol group, which indicates that ethanol can induce substantial damage to the colon as seen in both *in vitro* and *in vivo* models [58, 59]. Studies reported that administration of probiotic VSL#3 decreased the ethanol-induced epithelial permeability [60]. Also, supplementation of *L. plantarum* improves the colonic mucosal barrier dysfunction induced by the ethanol-fed diet [20]. The report showed reduced TEER and FITC-dextran hyperpermeability with Met treatment in a dextran sodium sulfate- (DSS-) induced colitis model [24]. In accordance with the above results, our results also demonstrated that coadministration of probiotic V and Met significantly prevented the decreased levels of TEER and intestinal hyperpermeability induced by ethanol indicating the combinatorial treatment completely blocks the ethanol-induced intestinal barrier dysfunction.

Several studies reported that alcohol and/or its metabolite (i.e., acetaldehyde) causes intestinal mucosal barrier dysfunction through alterations in TJ proteins [61, 62]. The interplay between ZO-1 and occludin is crucial for the assembly and preservation of TJs as well as the development of mucosal barrier function [63]. Occludin is the first identified and integral membrane TJ protein, which plays an important role in maintaining the barrier function and structural integrity of TJs [64]. Also, ZO-1 is a cytoskeletal linker protein that forms cross-links with other transmembrane proteins like occludins, necessary for the connection of other TJ proteins to the cytoskeleton [65]. The report demonstrated that the administration of 40 mM ethanol to Caco-2 monolayers disrupted the TJs, specifically occludins and ZO-1 [66]. Also, acute alcohol exposure disrupts the barrier function by downregulating the expressions of ZO-1 and occludin in colons of mice [18].

Supplementation of probiotic *L. plantarum* prevents the redistribution of TJ proteins (ZO-1 and occludin) from the colonic epithelial junction, which was induced by ethanol [20]. *A. muciniphila* showed protection against ethanol-induced gut leakiness by enhancing mucus thickness and TJ protein expression [67]. A scaffolding protein like tight junction protein-1 (TJP-1) is a gene that encodes for ZO-1 protein, an essential protein required for the maintenance of epithelial barrier integrity. The report proved that TJP-1 gene overexpression may be considered as an index of elevated intestinal barrier integrity in a VSL#3-induced NOD mice model [68]. Studies showed that pretreatment of *L. plantarum* ZLP001 markedly prevented the decreases in ZO-1 and occludin mRNA expression levels which is caused by enterotoxigenic *Escherichia coli* (ETEC) infection [69]. In both, Caco-2 monolayers and colitis mice model, Met administration provided protection against the decreased expression of occludin and ZO-1 as well as increased bacterial translocation [24, 70]. In Caco-2 monolayers, Met ameliorates the inhibiting effect of TNF-*α* on epithelial tight junction-related protein expression [71]. Therefore, the current study depicted that coadministration of probiotic V and Met ameliorates the dislocation of TJ proteins induced by alcohol in an *in vitro* and *in vivo* model of ethanol-induced intestinal barrier injury.

Alcohol metabolism through CYP2E1 may be a contributing factor towards the ethanol-induced intestinal effects. It is proven that alcohol products mediated through CYP2E1 could be the main inducers towards the ethanol-induced gut barrier dysfunction and hepatic injury. CYP2E1-mediated alcohol metabolism causes increased oxidative stress products like MDA that can generate reactive oxygen species/reactive nitrogen species (ROS/RNS) that can result in intestinal epithelial hyperpermeability or disruption [12, 13, 72]. A recent study showed increased CYP2E1 expression and activity in alcohol-induced Caco-2 monolayers and rodent models with gut leakiness [12]. Moreover, along with CYP2E1, there is also an activation of intestinal iNOS (also known as ROS-generating enzyme), which mediates ROS generation and, subsequently, mucosal barrier dysfunction induced by alcohol [73]. A recent study showed that individual as well as combined treatment of Met and probiotics significantly reduced ROS production in colorectal cancerous animals [74]. Reports showed that probiotics LC27 and LC67 may alleviate the GI inflammation by scavenging ROS [75]. Studies showed that Met treatment ameliorated the LPS-induced intestinal ROS production and oxidative stress [25]. Reports on Met showed decreased levels of TBARS and increased levels of antioxidant markers (GSH, SOD, and CAT) in colon tissue of the colitic rat model [76]. Current results depicting increased expression levels of CYP2E1 and iNOS along with colonic MDA resulted in the generation of ROS, which further resulted in alcohol-induced oxidative stress in rat colon. One result indicated a key finding of this study, i.e., combinatorial treatment of probiotic V and Met remarkably inhibit CYP2E1, thereby alleviating intestinal ROS generation and oxidative stress induced by alcohol.

To prevent oxidative stress, CYP2E1 also stimulates the expression of antioxidant factors like Nrf-2, which is considered to be one of the key transcription factors providing protection against oxidative stress/nitrative stress [77]. The transcription factor Nrf-2 is known to play an important role in the regulation of antioxidant gene expression [78]. In colonic mucosa, ethanol induced a decrease in the expression levels of IL-10 [63]. The corresponding changes in the expression of a few antioxidant genes by alcohol may be mediated by Nrf-2. Nrf-2 is a known regulator for the expression of genes like Gpx and NQO1. It is unknown whether catalase and SOD genes are regulated by Nrf-2 [63]. Studies demonstrated that probiotic *L. plantarum* inhibits ethanol-induced oxidative stress by upregulating the Nrf-2 expression [20]. Also, a recent study showed that Met significantly enhanced the nuclear translocation of Nrf-2 and its related target genes like HO-1 induced by LPS treatment [25]. In the colorectal cancer model, mixtures of genetically modified *lactobacilli* strains showed the curative anticancer effect through a combination of various anti-inflammatory machinery and IL-10 stimulation [79]. Interestingly, the present study conferred increased antioxidant activity of GSH-Px, SOD, CAT, Nrf-2, and HO-1 in the rats treated with probiotic V and Met in combination in the presence of ethanol when compared with either individual treatment of probiotic V or Met.

To further validate and understand the role of probiotic V and Met, we performed in silico analysis to check if the cotreatment of Met and probiotic V could act in synergism to prevent intestinal barrier injury. We explored the role of Met and butyrate in the activation of antioxidants like HO-1 and Nrf-2. As hepatic fibrosis in ALD has been shown to be mitigated by antioxidants like Nrf-2 and HO-1, their inducers can be promising agents to prevent fibrosis in the liver. Therefore, we checked if probiotic V and Met could synergistically induce the expression of Nrf-2 and HO-1. Upon analysis of their interactions in being able to induce Nrf-2 and HO-1, we found that both probiotic V and Met individually showed interactions with Nrf-2 and HO-1, with binding energies (Nrf-2: -6.0 and -3.4 kcal/mol; HO-1: -5.7 and -4.7 kcal/mol), which was significantly improved when docked in combination (Nrf-2: -9.7 kcal/mol; HO-1: -8.9 kcal/mol). This suggests that both probiotic V and Met could much better induce the expression of Nrf-2 and HO-1 when administered together as compared to their individual treatment. The *in silico* results are in agreement with the *in vitro* and *in vivo* results where the combination of Met and probiotic V induced the expression of Nrf-2 and HO-1.

ALD has been shown to alter lipid metabolism and increase the levels of serum triglycerides. Reports suggest that high circulating triglycerides also render men with a twofold risk of developing colorectal cancer [80]. Various reports also suggest that dyslipidemia can be associated with developing colorectal carcinoma [81]. Similarly, the present study also showed remarkably elevated levels of colonic TGs and cholesterol in the ethanol-fed rats, which was improved by treatment with probiotic V and Met cotreatment. Our previous study showed that combinatorial treatment of probiotic V and Met is known to prevent lipogenesis in ALD in the liver [26]; therefore, we explored its role in inhibiting alcohol-induced lipogenesis by regulating specific lipid metabolism-associated transcription factors in the intestine. In the hepatic tissue, unaided administration of Met and probiotic V showed AMPK activation, which is otherwise inhibited in the presence of ethanol confirming its therapeutic role in lipid metabolism [26]. The current study depicted that coadministration of probiotic V and Met activates AMPK and meanwhile inhibits the expression of SREBP-1c, thereby ameliorating ethanol-induced lipogenesis. With the alteration in expression of SREBP-1c, it further deactivated the downstream signaling cascades, viz., lipogenic enzymes like ACC and FAS, overall improving the ethanol-induced intestinal barrier injury.

As discussed, ethanol metabolism involving CYP2E1 causes lipid peroxidation, and further, it forms protein adducts as a result of the end product of lipid peroxidation. This also affects the ER functioning and protein folding. In the ER, there is an accumulation of protein adducts which causes ER fragmentation [82]. The resulting misfolded proteins lead to the upregulated mRNA expression levels of ER stress-related transcription factor-like CHOP and protein-like Grp78 in alcohol-fed mice. In colonic tissues, studies reported improved ER stress which was relatively responsible for the improved inflammation in colitis-infected mice [83]. Also, ethanol consumption upregulated the levels of CHOP and Grp78 in Caco-2 colon cancer cells [84]. No reports have depicted the role of Met in attenuating ER stress in colonic mucosa. Therefore, our report was the first one to depict the role of Met alone as well as in combination with probiotic V in attenuating the ethanol-induced ER stress in colonic mucosa. The present study also showed that probiotic V and Met in combination is capable of reducing the expression levels of ER stress genes (CHOP and Grp78) more significantly in the presence of ethanol when compared with the individual treatment of probiotic V or Met.

In the past decade, breakthrough inflammation research reported that intestinal inflammation and oxidative stress can cause a leaky gut barrier or hyperpermeability in the intestinal tract. In addition, a transgenic model with a leaky gut showed manifestation of intestinal inflammation [85]. Intestinal inflammation is mostly connected with mucosal barrier dysfunction and TJ disruption [25]. It is known that alcohol-induced oxidative stress caused mucosal barrier dysfunction and epithelial TJ disruption, which resulted in luminal LPS leakage in the colonic mucosa. Ingestion of alcohol upregulates the myeloperoxidase activity in the colon [75]. Recent reports demonstrated the synergistic effect of Met and probiotic in reducing the levels of TNF-*α* and IL-6 in the colon and serum resulting in a marked improvement in the inflammatory response [74]. Studies showed that probiotic *L. plantarum* downregulated the expression levels of cytokines (TNF-*α*, IL-6, and IL-*β*) in the colonic mucosa, thereby preventing the ethanol-induced inflammatory response [20]. The study demonstrated that VSL#3 administration induces a remarkable reduction in cytokine expression, i.e., IL-1*β* in the intestinal mucosa of the NOD mice [68]. The report showed that Met treatment could suggestively prevent LPS-induced proinflammatory cytokines like IL-6 and TNF-*α* expression in the mice model of intestinal tissue [25]. In agreement with the above reports, the present study also demonstrated the significantly increased mRNA expression levels of TNF-*α*, IL-6, and IL-*β* in the ethanol group compared to the control in an *in vivo* and *in vitro* model. The present study demonstrated the downregulated expression of TNF-*α*, IL-6, and IL-*β* indicating the anti-inflammatory effect of combinatorial treatment of probiotic V and Met in the *in vitro* as well as *in vivo* model.

It has been reported that a consortium of eight bacterial strains, i.e., VSL#3 (similar to Visbiome®), was shown to increase the levels of SCFA butyrate with no changes in other SCFAs [86]. Butyrate is considered to be a prime energy source for colonocytes and also helps in regulating intestinal homeostasis through anti-inflammatory mechanisms. In accordance with the above studies, the current study demonstrated the increased levels of butyrate in ethanol-fed rats treated with probiotic V. Therefore, it is of prime importance to understand how butyrate has been absorbed and transported to the intestine. Several studies showed that the butyrate receptor, i.e., GPR109A, and the butyrate transporter, i.e., SLC5A8, are required for the same, and their expressions are circumscribed to the apical membranes of intestinal and colonic epithelial cells [87, 88], thus permitting uninterrupted access to butyrate in the lumen. SLC5A8 is a butyrate transporter linked with Na^+^ required for the butyrate transport into cells [89]. GPR109A is a butyrate receptor that is intricated in the stimulation of surface expression and activity of butyrate transporters. These synchronized regulatory mechanisms eventually led to increased absorption of butyrate when elevated concentrations of butyrate are accessible [90]. Alcohol consumption as discussed earlier disrupts intestinal permeability by reducing the expression of TJ proteins. The restoration of intestinal permeability can be of higher therapeutic benefit to prevent the progression of ALD. The SCFA butyrate has been shown to promote the epithelial barrier function by upregulating the expression of genes encoding TJ constituents and protein reassembly through STAT3 and SP1 activation [91]. The present study showed that chronic alcohol consumption led to the downregulated expression of butyrate sensors, i.e., GPR109A and SLC5A8 proteins in the rat colon, while the cotreatment of probiotic V and Met prevented these changes in ethanol-fed rats.

As discussed earlier, butyrate plays a very important role in maintaining intestinal permeability, but the absorption of SCFAs is highly enhanced by SLC5A8, and SCFAs are shown to activate signaling pathways via GPR109A. Considering the role of GPR109A as the butyrate receptor and SLC5A8 as the butyrate transporter, we explored the role of Met and butyrate in interacting with GPR109A and SLC5A8 by in silico analysis. As expected, being the ligand for both proteins, butyrate showed a good binding affinity with GPR109A and SLC5A8. The main aim of our study was to see if butyrate and Met could work in synergism or enhance the affinity of butyrate towards its receptor. Therefore, we docked both Met and butyrate simultaneously, and we found that the cotreatment of both the ligands enhanced the binding affinities (GRP109A: -9.7 kcal/mol; SLC5A8: -8.3 kcal/mol) as compared to their individual binding affinities (GRP109A: -4.9 and -3.4 kcal/mol; SLC5A8: -5.1 and -4.7 kcal/mol) suggesting their synergism in activating GPR109A and SLC5A8. The *in silico* results are in agreement with the *in vitro* and *in vivo* results where the combination of Met and probiotic V induced the expression of GPR109A and SLC5A8.

## 5. Conclusion

**Figure 20:**
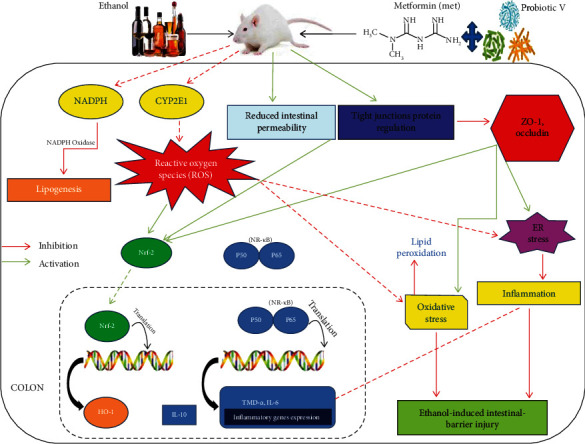
Potential preventive mechanism of probiotic V and Met in combination against ethanol-induced intestinal barrier injury.

The current study stipulated the confirmation for the potential role of oxidative stress and inflammatory response in ethanol-induced disruption of colonic TJs, intestinal hyperpermeability, and endotoxemia both *in vitro* and *in vivo* and that the supplementation of probiotic V and Met prevents alcoholic-induced gut integrity and permeability, endotoxemia, lipogenesis, inflammatory responses, and ROS generation and upregulates the antioxidant gene expression (Figure 20). We further verify that supplementation of probiotic V and Met effectively prevents ethanol-induced intestinal barrier dysfunction by acting on butyrate receptors and transporters. For the very first time, we report here the combined protective function of probiotic V and Met in the human intestinal epithelial barrier. In addition, also for the first time, the current study showed how the probiotic V and Met could act in synergism by demonstrating the *in silico* interactions of both ligands in inducing the expressions of antioxidant machinery like HO-1 and Nrf-2 and also the butyrate receptor GPR109A and transporter SLC5A8, which matched with our findings, further validating our hypothesis. Such knowledge will provide support for developing the combination of probiotic V and Met as a therapeutic candidate to improve ethanol-induced intestinal epithelial barrier dysfunction or gut leakiness. Additionally, this may provide a strong piece of evidence for further prospective research which will unravel its potential application in humans.

## Figures and Tables

**Table 1:** The list of *Homo sapiens* primers used for the quantification of mRNA expression levels in qRT-PCR.

Sr. no.	Name of the gene	Forward primer sequence	Reverse primer sequence
1	18S	ACGGAAGGGCACCACCAGGA	CACCACCACCCACGGAATCG
2	ZO-1	TATTATGGCACATCAGCACG	TGGGCAAACAGACCAAGC
3	Occludin	CTCGAGAAAGTGCTGAGTGCCTGGAC	AAGCTTTCGGTGACCAATTCACCTGA
4	CYP2E1	AGGGTACCATGTCTGCCCTCGGAGTGA	ACAATTTGAAAGCTTGTTTGAAAGCGG
5	NOX	GTACAAATTCCAGTGTGCAGACCAC	GTACAAATTCCAGTGTGCAGACCAC
6	TNF-*α*	CCCTCACACTCAGATCATCTTCT	GCTACGACGTGGGCTACAG
7	IL-6	GACAACTTTGGCATTGTGG	ATGCAGGGATGATGTTCTG
8	IL-10	ACTGCTAACCGACTCCTTA	TAAGGAGTCGGTTAGCAGT

**Table 2:** The list of *Rattus norvegicus* primers used for the quantification of mRNA expression levels in qRT-PCR.

Sr. no.	Name of the gene	Forward primer sequence	Reverse primer sequence
1	18S	GTTGGTTTTCGGAACTGAGGC	GTCGGCATCGTTTATGGTCG
2	ZO-1	TCGGAGCTCGGGCATTATTC	CAGGGCACCATACCAACCAT
3	Occludin	CAACGGCAAAGTGAATGGCA	CTTTCCCCTTCGTGGGAGTC
4	Nrf-2	CAGAGTTTCTTCGCCAGAGG	TGAGTGTGAGGACCCATCG
5	HO-1	CAAATCCCACCTTGAACACA	CGACTGACTAATGGCAGCAG
6	CYP2E1	TCAATCTCT GGACCCCAACTG	GCGCTCTGCACTGTGCTTT
7	NOX	TGACAGTGATGTATGCAGCAT	CAGCTTGTTGTGTGCACGCTG
8	CHOP	ACCACCACACCTGAAAGCA	AGCTGGACACTGTCTCAAAGG
9	Grp78	CCGTAACAATCAAGGTCTACGA	AAGGTGACTTCAATCTGGGGTA
10	TNF-*α*	TCTCATTCCTGCTCGTGGCG	GGTGAGGAGCACGTAGTCGG
11	IL-6	TTGACAGCCACTGCCTTCCC	CGGAACTCCAGAAGACCAGAGC
12	IL-10	TGCCTTCAGTCAAGTGAAGAC	AAACTCATTCATGGCCTTGTA
13	AMPK	GCTGTGGATCGCCAAATTAT	GCATCAGCAGAGTGGCAATA
14	SREBP-1c	TCTGCCTTGATGAAGTGTGG	AGCAGCCCCTAGAACAAACA
15	FASN	CCTCAGTCCTGTTATCACCCGA	GCTGAATACGACCACGCACTA
16	ACC	CCTTCTTCTACTGGCGACTGAG	TAAGCCTTCACTGTGCCTTCC
17	GPR109A	ACTTTCTGGTGATAAACGGCAAGA	GACTGTCAGGCCGATGGTG
18	SLC5A8	AGCCAGCACTCAGCGTATTT	TTTGAGCTCCAATTCCAACC

**Table 3:** Binding energies and amino acid interaction for Met and butyrate on performing molecular docking with respective protein structures.

Protein	Ligand	Binding energy (kcal/mol)	Amino acid interactions
Nrf-2	SLLD Met	-6.0	ILE: 559, VAL: 606, GLY: 367, GLY: 418, VAL: 512, VAL: 465
	SLLD butyrate	-3.4	GLY: 605, ILE: 559, GLY: 367, VAL: 606
	MLSD Met and butyrate	-9.7	ILE: 559, GLY: 367, GLY: 558, VAL: 463, VAL: 604, LEU: 365, LEU: 557
HO-1	SLLD Met	-5.7	TYR: 286, ILE: 419, SER: 418, GLU: 565, HIS: 620, GLU: 623
	SLLD butyrate	-4.7	ALA: 272, SER: 267, GLN: 268, TYR: 321, THR: 269
	MLSD Met and butyrate	-8.9	PHE: 699, THR: 188, THR: 703, LYS: 700, HIS: 803, GLU: 402, GLU: 623, LEU: 624, LEU: 625
GPR109A	SLLD Met	-4.9	ASN: 321, ASN: 320, ARG: 308, SER: 323, THR: 324, ARG: 311
	SLLD butyrate	-3.4	ILE: 162, ALA: 142, ASN: 65, PHE: 64, TRP: 56
	MLSD Met and butyrate	-9.7	ILE: 162, ALA: 142, ASN: 65, PHE: 64, TRP: 56, ASN: 321, ASN: 320, ARG: 308, SER: 323, THR: 324, ARG: 311
SLC5A8	SLLD Met	-5.1	GLN: 249, PHE: 458, GLY: 462
	SLLD butyrate	-4.7	GLN: 82, THR: 63
	MLSD Met and butyrate	-8.3	GLN: 82, THR: 63, VAL: , 281, GLN: 249, GLY: 412, GLY: 242

## Data Availability

The authors confirm that the data supporting the findings of this study are available within the article.

## Supplementary Materials

Supplementary Materials Supplementary Material: multiple sequence alignment (MSA) and the Ramachandran plot of Nrf-2, GRP109A, and SLC5A8. Supplementary Figure 1: multiple sequence alignment (MSA) was performed between target protein nuclear factor erythroid 2-related factor (NRF-2) of *Rattus norvegicus* (accession no. O54968) and the template protein Kelch-like ECH-associated protein 1 of *Mus musculus* (mouse) (accession no. Q9Z2X8). Due to the unavailability of the target protein in PDB, the modeled protein for the same was built using the structure of the template protein. MSA was performed to establish a sense of conservedness among both proteins and to justify the selection of templates per se. Observed identity is 75.9% and similarity is 24.6% among both the sequences. Supplementary Figure 2: the Nrf-2 Ramachandran plot results were performed using SAVES server v6.0 PROCHECK. Supplementary Figure 3: GPR109A—multiple sequence alignment (MSA) was performed between target protein hydroxycarboxylic acid receptor 2 of *Rattus norvegicus* (accession no. Q80Z39) and the template protein G-protein-coupled receptor APJ (apelin receptor) of *Homo sapiens* (accession no. P35414). Due to the unavailability of the target protein in PDB, the modeled protein for the same was built using the structure of the template protein. MSA was performed to establish a sense of conservedness among both proteins and to justify the selection of templates per se. Observed identity is 97.8% and similarity is 21.8% among both the sequences. Supplementary Figure 4: the GPR109A Ramachandran plot results were performed using MolProbity. Supplementary Figure 5: quality checks on the parameters for the GPR109A modeled protein were performed using the SWISS MODEL. Supplementary Figure 6: SLC5A8—multiple sequence alignment (MSA) was performed between target protein electrogenic sodium monocarboxylate cotransporter of *Rattus norvegicus* (accession no. D3Z9E5) and the template protein putative sodium : solute symporter of *Proteus mirabilis* (accession no. B4EZY7). Due to the unavailability of the target protein in PDB, the modeled protein for the same was built using the structure of the template protein. MSA was performed to establish a sense of conservedness among both proteins and to justify the selection of templates per se. Observed identity is 75.9% and similarity is 24.6% among both the sequences. Supplementary Figure 7: the SLC5A8 protein Ramachandran plot results were performed using MolProbity. Supplementary Figure 8: quality check parameters for the SLC5A8 modeled protein were performed using the SWISS MODEL.
