# Supplementary material for: Prophylactic Treatment of Probiotic and Metformin Mitigates Ethanol-Induced Intestinal Barrier Injury: In Vitro, In Vivo, and In Silico Approaches
Source: Mediators Inflamm. 2021 Sep 27;2021:5245197. doi: 10.1155/2021/5245197 (PMC8490080; doi:10.1155/2021/5245197)
Supplement: Supplementary Materials — Supplementary Material: multiple sequence alignment (MSA) and the Ramachandran plot of Nrf-2, GRP109A, and SLC5A8. Supplementary Figure 1: multiple sequence alignment (MSA) was performed between target protein nuclear factor erythroid 2-related factor (NRF-2) of Rattus norvegicus (accession no. O54968) and the template protein Kelch-like ECH-associated protein 1 of Mus musculus (mouse) (accession no. Q9Z2X8). Due to the unavailability of the target protein in PDB, the modeled protein for the same was built using the structure of the template protein. MSA was performed to establish a sense of conservedness among both proteins and to justify the selection of templates per se. Observed identity is 75.9% and similarity is 24.6% among both the sequences. Supplementary Figure 2: the Nrf-2 Ramachandran plot results were performed using SAVES server v6.0 PROCHECK. Supplementary Figure 3: GPR109A—multiple sequence alignment (MSA) was performed between target protein hydroxycarboxylic acid receptor 2 of Rattus norvegicus (accession no. Q80Z39) and the template protein G-protein-coupled receptor APJ (apelin receptor) of Homo sapiens (accession no. P35414). Due to the unavailability of the target protein in PDB, the modeled protein for the same was built using the structure of the template protein. MSA was performed to establish a sense of conservedness among both proteins and to justify the selection of templates per se. Observed identity is 97.8% and similarity is 21.8% among both the sequences. Supplementary Figure 4: the GPR109A Ramachandran plot results were performed using MolProbity. Supplementary Figure 5: quality checks on the parameters for the GPR109A modeled protein were performed using the SWISS MODEL. Supplementary Figure 6: SLC5A8—multiple sequence alignment (MSA) was performed between target protein electrogenic sodium monocarboxylate cotransporter of Rattus norvegicus (accession no. D3Z9E5) and the template protein putative sodium : solute symporter of Proteus [file 5245197.f1.docx]

**SUPPLEMENTARY FIGURES**


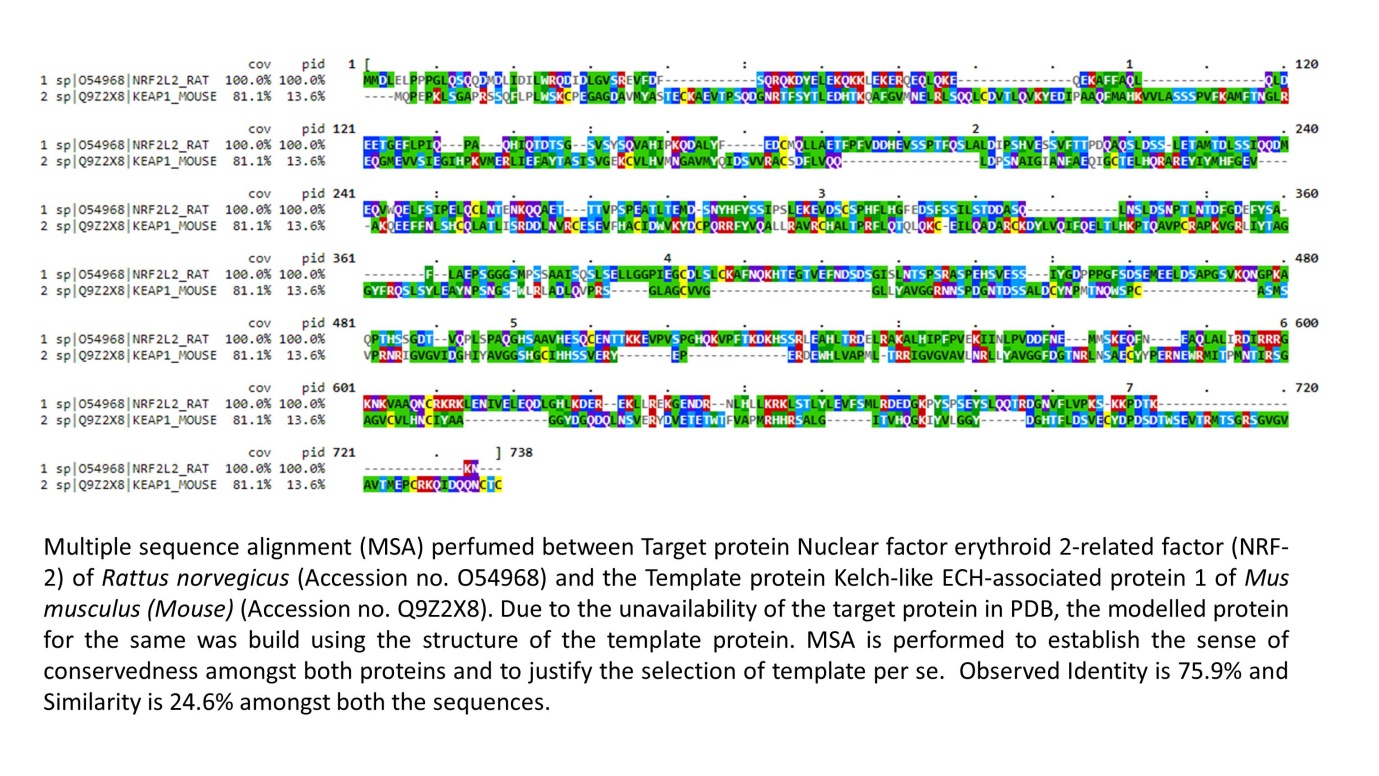


**Supplementary Figure 1:** Multiple sequence alignment (MSA) perfumed between Target protein Nuclear factor erythroid 2-related factor (NRF-2) of *Rattus norvegicus* (Accession no. O54968) and the Template protein Kelch-like ECH-associated protein 1 of Mus musculus (Mouse) (Accession no. Q9Z2X8). Due to the unavailability of the target protein in PDB, the modeled protein for the same was build using the structure of the template protein. MSA is performed to establish the sense of conservedness amongst both proteins and to justify the selection of templates per se. Observed Identity is 75.9% and Similarity is 24.6% amongst both the sequences.


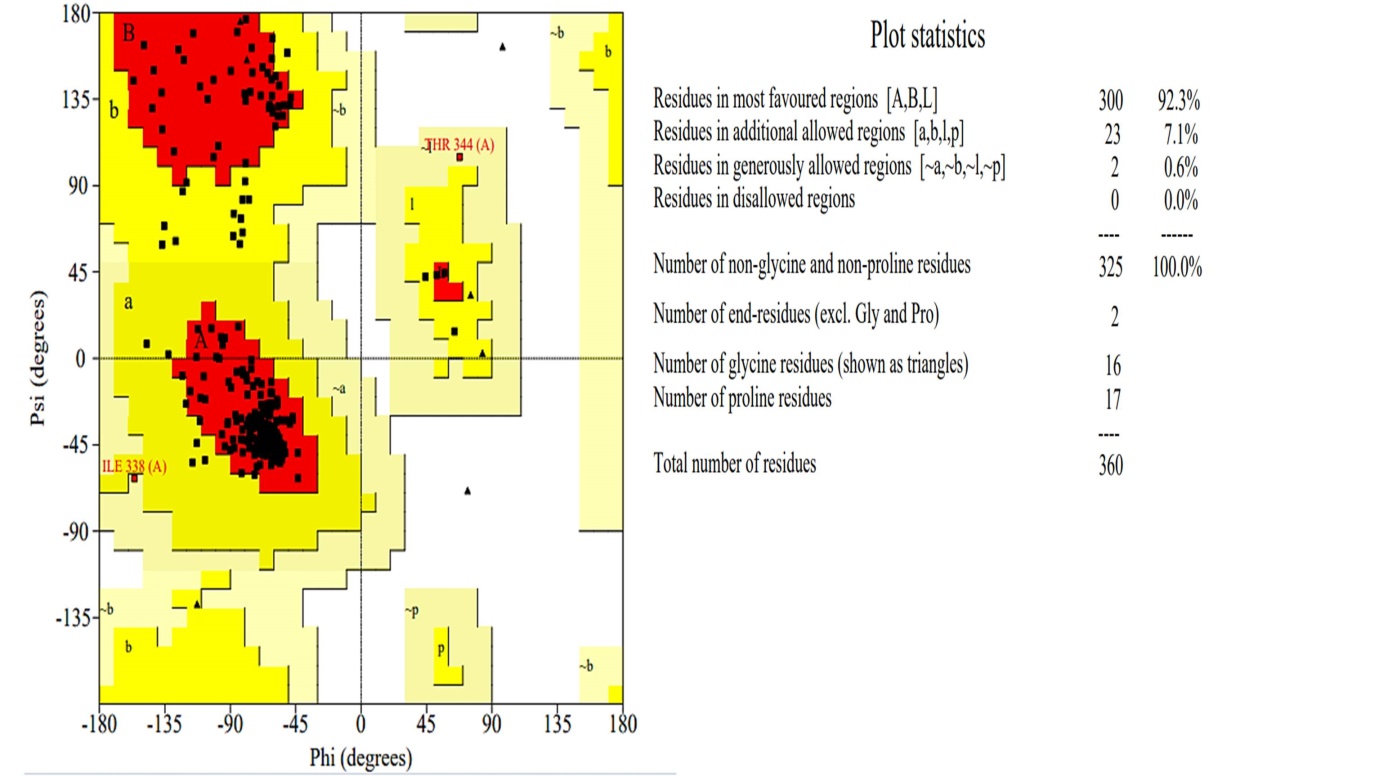


**Supplementary Figure 2:** Nrf-2 Ramachandran Plot results performed using SAVES server v6.0 PROCHECK.


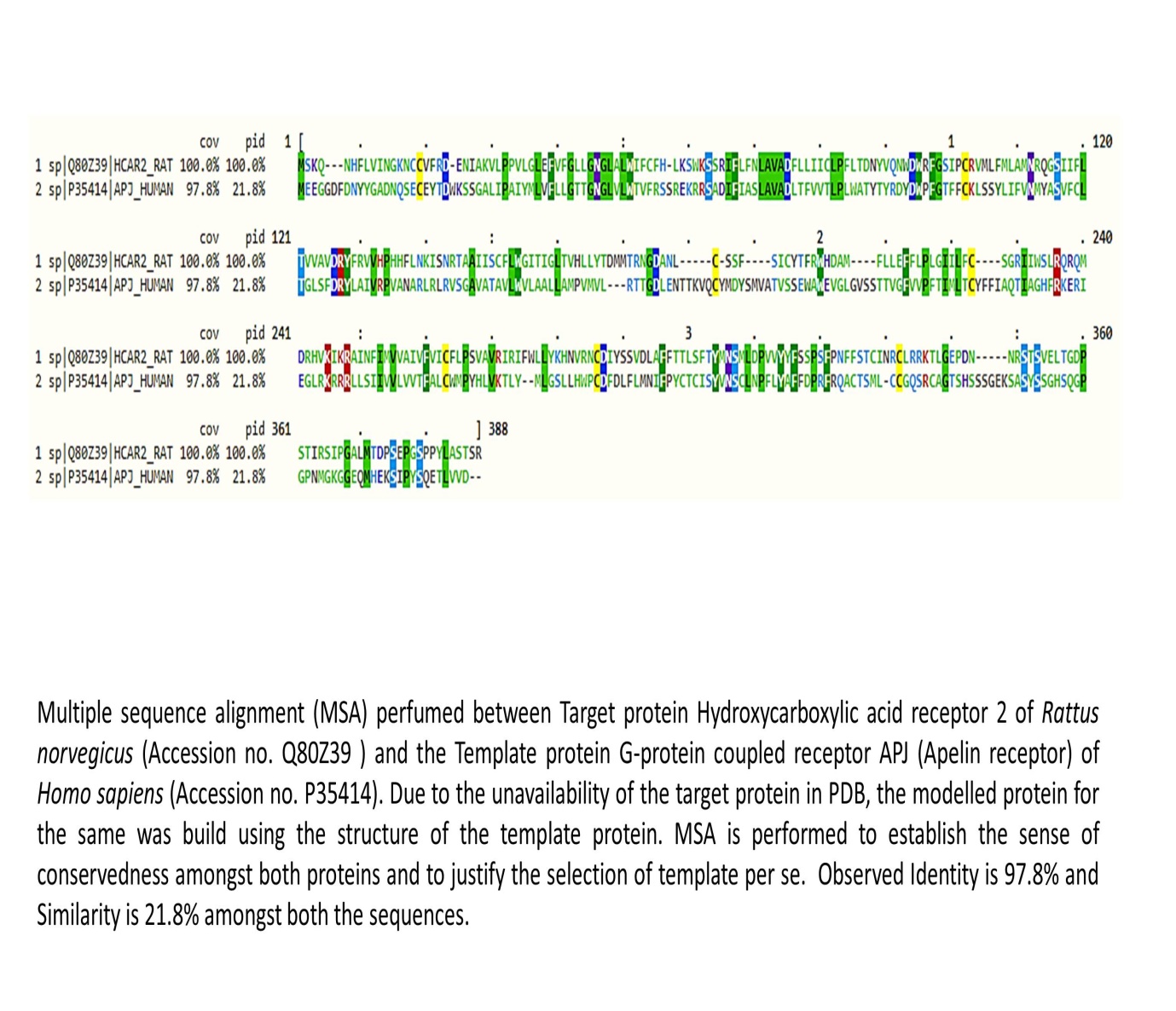


**Supplementary Figure 3:** GPR109A: Multiple sequence alignment (MSA) perfumed between Target protein Hydroxycarboxylic acid receptor 2 of *Rattus norvegicus* (Accession no. Q80Z39) and the Template protein G-protein coupled receptor APJ (Apelin receptor) of Homo sapiens (Accession no. P35414). Due to the unavailability of the target protein in PDB, the modeled protein for the same was build using the structure of the template protein. MSA is performed to establish the sense of conservedness amongst both proteins and to justify the selection of templates per se. Observed Identity is 97.8% and Similarity is 21.8% amongst both the sequences.


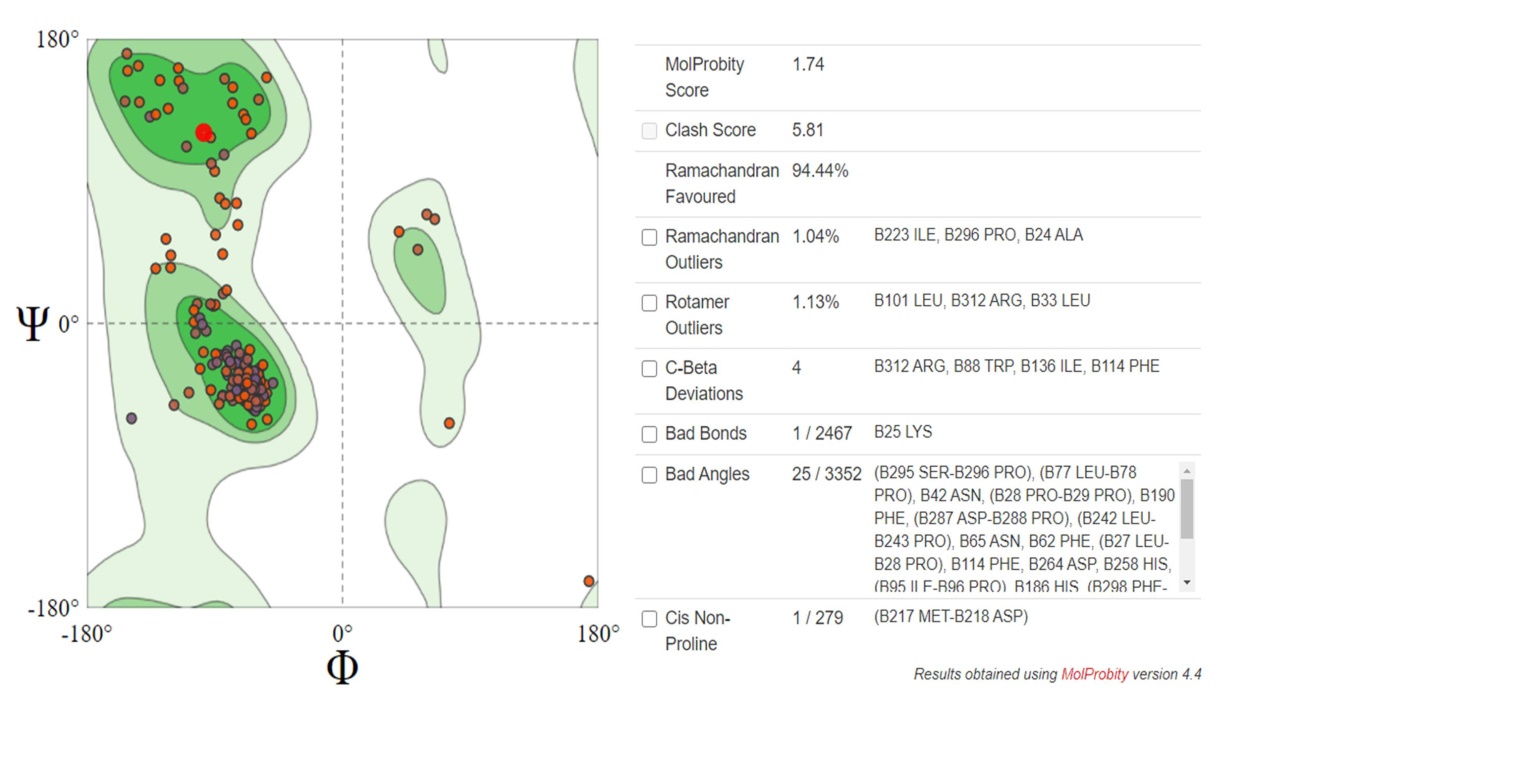


**Supplementary Figure 4:** GPR109A Ramachandran Plot results performed using MolProbity.


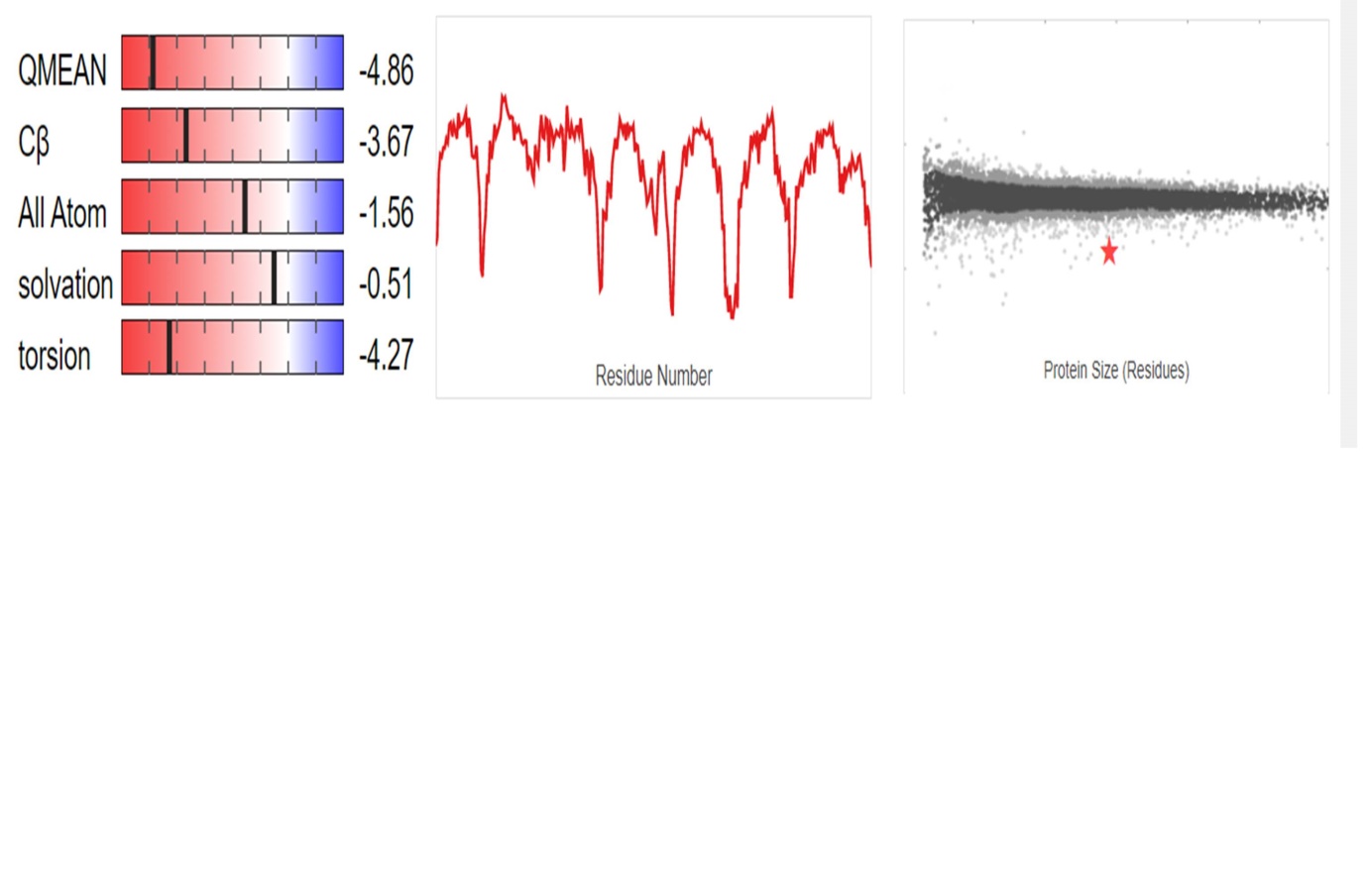


**Supplementary Figure 5:** Quality check parameters for GPR109A Modelled Protein performed using Swiss Model.

**
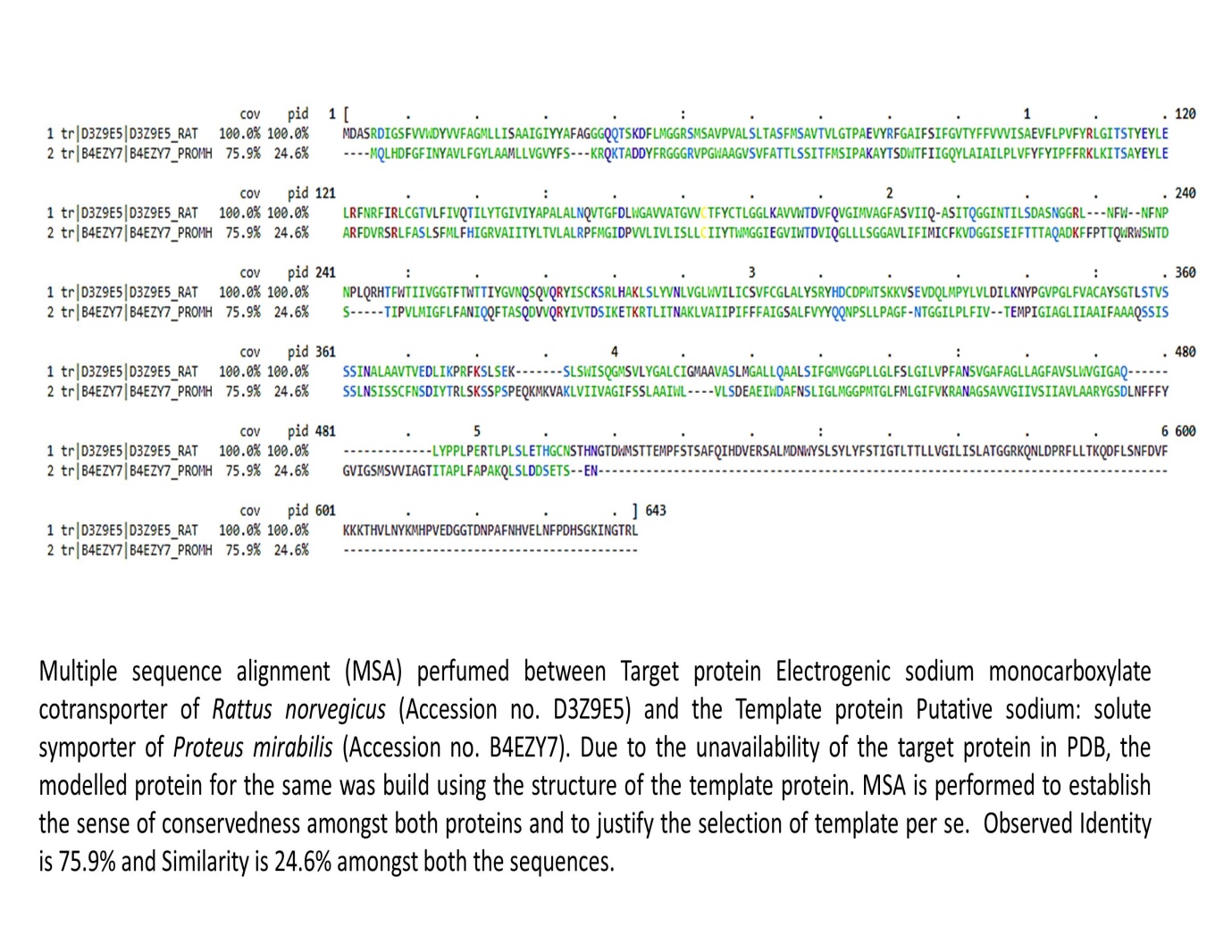
**

**Supplementary Figure 6:** SLC5A8: Multiple sequence alignment (MSA) perfumed between Target protein Electrogenic sodium monocarboxylate cotransporter of *Rattus norvegicus* (Accession no. D3Z9E5) and the Template protein Putative sodium: solute symporter of Proteus mirabilis (Accession no. B4EZY7). Due to the unavailability of the target protein in PDB, the modeled protein for the same was build using the structure of the template protein. MSA is performed to establish the sense of conservedness amongst both proteins and to justify the selection of templates per se. Observed Identity is 75.9% and Similarity is 24.6% amongst both the sequences.


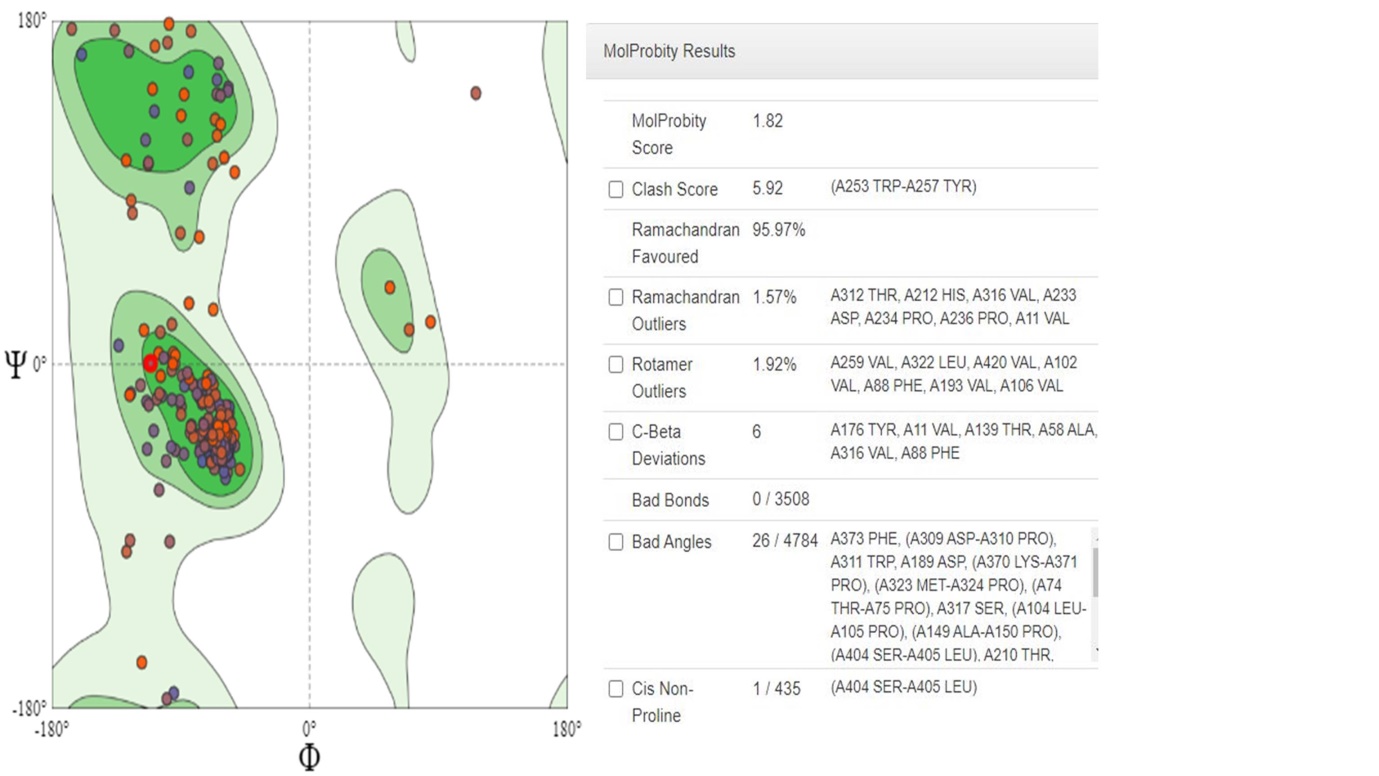


**Supplementary Figure 7:** SLC5A8 protein Ramachandran plot results performed using MolProbity.

**
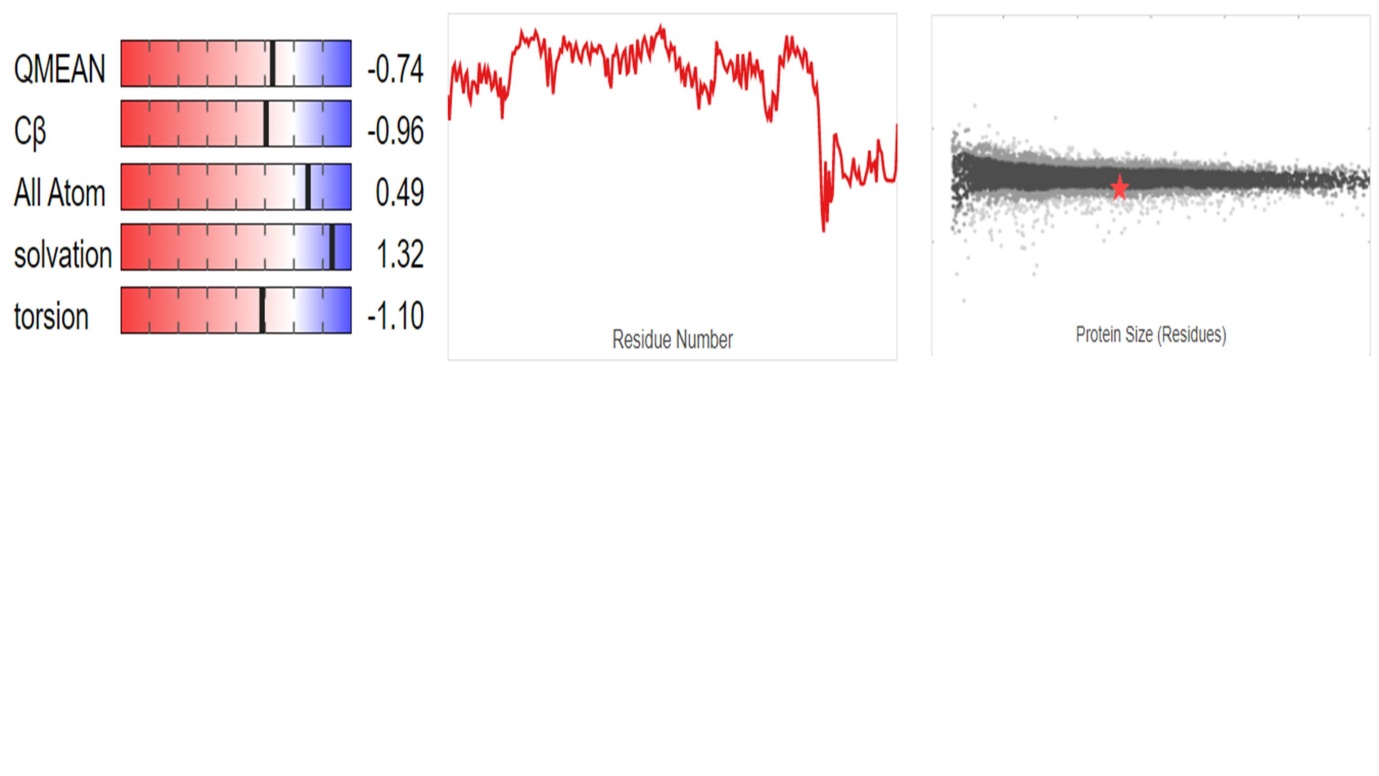
**

**Supplementary Figure 8:** Quality check parameters for SLC5A8 Modelled Protein performed using Swiss Model.
